# Ligand-Engineered Methylammonium Lead Bromide Nanoplatelets: Single-Photon Emission and Strong Light-Matter Coupling

**DOI:** 10.1021/acsnano.6c01048

**Published:** 2026-03-31

**Authors:** Taras V. Sekh, Taehee Kim, Sebastian Sabisch, Federica Bertolotti, Laura Caliò, Juan F. Galisteo-López, Francisco J. Coto-Ruiz, Lucía Santiago-Andrades, Antonietta Guagliardi, Norberto Masciocchi, Rolf Erni, Hernán Miguez, Gabriele Rainò, Maryna I. Bodnarchuk, Maksym V. Kovalenko

**Affiliations:** † Institute of Inorganic Chemistry, Department of Chemistry and Applied Biosciences, 27219 ETH Zürich, Zürich 8093, Switzerland; ‡ Laboratory for Thin Films and Photovoltaics, EMPA−Swiss Federal Laboratories for Materials Science and Technology, Dübendorf 8600, Switzerland; § Department of Science and High Technology and To.Sca.Lab, 19045 University of Insubria, via Valleggio 11, Como 22100, Italy; ∥ Instituto de Ciencia de Materiales de Sevilla, Consejo Superior de Investigaciones Científicas-Universidad de Sevilla, Sevilla 41092, Spain; ⊥ Istituto di Cristallografia and To.Sca.Lab, Consiglio Nazionale delle Ricerche, via Valleggio 11, Como 22100, Italy; # Electron Microscopy Center, 28501 Empa−Swiss Federal Laboratories for Materials Science and Technology, Dübendorf CH-8600, Switzerland

**Keywords:** nanoplatelets, lead halide perovskites, ligands, assemblies, photoluminescence, polaritons

## Abstract

Lead halide perovskite nanoplatelets (LHP NPLs) are of immense interest in the materials science and optoelectronics communities owing to their strong quantum confinement leading to narrow emission peaks, thickness-dependent tunable photoluminescence, and large exciton binding energies. Thus far, their further development and photophysical investigations at ensemble and single-particle levels have been impeded by suboptimal ligand passivation, inferior environmental and colloidal stability compared to their 3D nanocrystal counterparts, and limited compositional diversity. Here, we report highly monodisperse methylammonium lead bromide (MAPbBr_3_) NPLs (11.3 ± 2.3 × 1.7 ± 0.5 nm) with tunable ligand chemistry and enhanced photoluminescence quantum yields of up to 80%. NPLs capped with zwitterionic ligands exhibit improved stability upon air exposure and sequential purification. Nuclear magnetic resonance spectroscopy on ligand-exchanged NPLs, capped with either phosphocholine- or phosphoethanolamine-type ligands, confirms the partial replacement of the pristine ligands. Owing to the size and shape uniformity of synthesized MAPbBr_3_ NPLs, they readily form assemblies of stacked face-to-face NPLs, as observed in both colloidal dispersions and films, giving rise to concentration-dependent multicolor emission. The temperature dependence of the NPL emission exhibits a nonmonotonous trend, attributed to the highly anisotropic confinement and the consequent exciton–phonon coupling. We also observed photoluminescence from single MAPbBr_3_ NPLs at both room and cryogenic temperatures, revealing highly polarized fine-structure emission lines with single-photon purity exceeding 80%. The high uniformity and optical transparency of MAPbBr_3_ NPL films enabled their integration into optical cavities, where they exhibited strong light-matter coupling with a substantial Rabi splitting of 200 meV.

## Graphical abstract

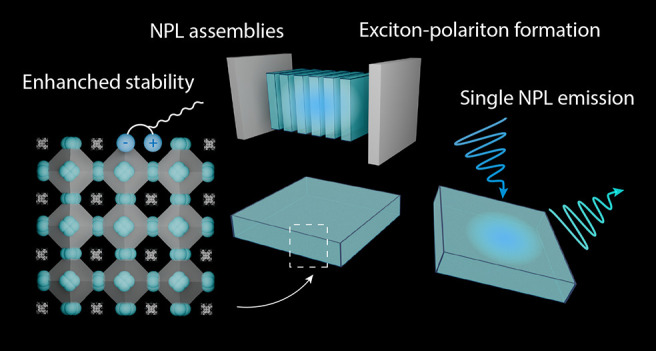

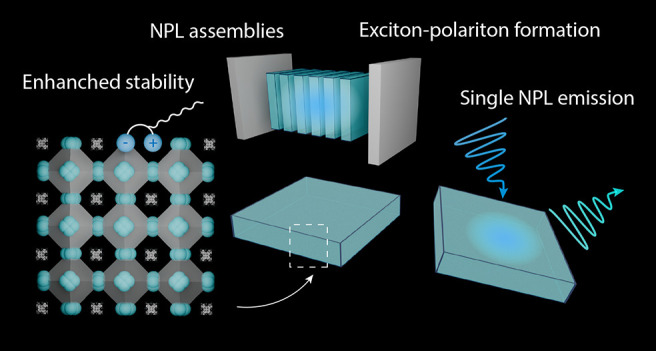

Atomically flat colloidal lead halide perovskite (LHP) nanoplatelets (NPLs) are highly sought after as versatile excitonic materials for photonics and optoelectronics.^1–10^ Strong quantum and dielectric confinement arises from the extreme thickness reduction, down to one octahedron,^11–14^ pushing the emission into the deep-blue spectral range, otherwise accessible only via mixed bromide-chloride nanocrystals (NCs)^15–17^ or sub-5 nm LHP NCs,^18–21^ suffering from challenging synthesis and poor stability.

Exquisite control over all three morphological parameters of cuboidal NCs (cubes, rods, platelets) is crucial for tailoring their excitonic properties, as well as for the subsequent mesostructural order in their dense assemblies, wherein a high degree of orientational and positional ordering fosters the emergence of collective properties in these solids, such as strongly directed emission,^22^ efficient FRET transfer in CdSe NPLs,^23–25^ and stimulated emission.^26–29^ Ligand-assisted reprecipitation (LARP) synthesis,^30^ combined with long-chain ammonium ligands, yielded a uniform and tunable LHP NPL thickness, governed by the precursor ratio and alkylamine chain length.^12,14,31,32^ Early LARP syntheses primarily reported the formation of laterally extended, μm-sized platelets with limited colloidal processability and poor emissivity.^12–14^ Likewise, in the realm of II–VI materials, early works yielded extended CdSe nanosheets and nanoribbons,^33–36^ which then synthetically evolved into laterally controlled NPLs.^37–39^ This advancement was followed by the engineering of epitaxial heterostructures and advances in photophysics,^40–43^ such as low-threshold lasing and single-NPL spectroscopic characterization.^26–29,44–46^

The ability to precisely control all three nanocuboid dimensions is equally crucial for LHPs. The main hurdle to obtaining and retaining 3D-precise LHP NCs (i.e., with all three dimensions tunable and with low dispersion), especially for thin NPLs, is their structural softness, which arises from their much lower lattice energy relative to that of II–VI semiconductor NCs.^47^ Available studies mainly concern CsPbX_3_ NPLs,^1–3,48,49^ affording ensemble-level optical characterization of emission anisotropy and excitons. Rare single-particle studies demonstrated high single-photon purity at cryogenic temperatures and exciton fine-structure splitting.^50–52^ To our knowledge, reports presenting narrow dispersion of all three cuboid parameters for anisotropic CsPbBr_3_ NCs are limited; only a few examples exist for NPLs^1,53^ and nanorods,^54,55^ contrasting the abundance of publications on near-monodisperse near-cubic NCs of all LHP compositions. Transition to shape-engineered anisotropic NCs of hybrid organic–inorganic LHPs, with methylammonium (MA) or formamidinium (FA) as A-site cations, is further aggravated by the chemical instabilities of these compositions.^56^ To date, even isotropic sub-5 nm MAPbBr_3_ and FAPbBr_3_ NCs remain elusive.

The other Achilles’ heel of LHP NCs is their inherently labile surface chemistry. The selection of ligands cannot invoke head groups forming stronger bonds with LHP constituents than the bonds within the soft LHP lattice.^47^ The ammonium-based ligands, typically employed for LHP NPL growth, exhibit dynamic binding, thereby compromising the retention of the colloidal state and structural integrity during subsequent purification and processing.^57^ To date, surface modification of LHP NPLs has chiefly been limited to CsPbBr_3_ NPLs combined with phosphonates,^58^ sulfonates,^49,59^ and sulfates.^60^ An emerging class of formally neutral yet more statically binding ligands comprising multiple binding sites—zwitterionic ligands^61–63^ —have thus far not been thoroughly tested for LHP NPLs.

In this work, we report the synthesis of highly monodisperse MAPbBr_3_ NPLs with lateral dimensions of 11.3 ± 2.3 nm and a thickness of 1.7 ± 0.5 nm, followed by a postsynthetic ligand exchange of the growth-mediating ammonium ligands with the latest generation of phosphoethanolamine and phosphocholine zwitterionic ligands. The enhanced surface passivation resulted in photoluminescence quantum yields (PLQYs) of up to 80%. 2-ammonioethyl 2-octyl-1-dodecyl phosphate (C_8_C_12_–PEA)- and lecithin-capped NPLs retain colloidal integrity and emissivity for months under ambient conditions. X-ray diffraction analysis indicates that MAPbBr_3_ NPLs exhibit a tetragonal distortion of the cubic unit cell along the thickness direction. MAPbBr_3_ NPLs readily self-assemble in a face-to-face manner into sub-μm-sized superlattices (SLs), both in the solid state and colloidal solution. At cryogenic temperatures, the ensemble of MAPbBr_3_ NPLs displayed sharp excitonic emission at 2.7 eV, along with a broader, red-shifted feature, originating from excimer/trion emission of NPL assemblies, which disappears at lower NPL concentrations. Unlike nanocubes of comparable volume, MAPbBr_3_ NPLs exhibited a nonmonotonic temperature dependence of PL emission, attributed to the interplay between thermal expansion and electron–phonon coupling. Upon cooling, single NPLs exhibit a narrowing of the emission line width, from 90 meV at room temperature (RT) down to 11 meV at 4 K, highly polarized emission, and pronounced antibunching behavior with a g^(2)^(0) of 0.2, making them appealing candidates as single-photon emitters. The facile fabrication of nonscattering NPL films with high uniformity enabled the integration of MAPbBr_3_ NPLs into Fabry-Pérot resonators. As a result of strong light-matter coupling inside the optical cavity, at room temperature, both the absorption and emission measurements revealed polariton formation with a Rabi splitting of 200 meV—a value far exceeding that reported for other LHP NCs.^64,65^

## Results and Discussion

### Synthesis of MAPbBr_3_ NPLs

The synthesis of MAPbBr_3_ NPLs was motivated by the report on nanoclusters,^66^ wherein malic acid (MLA) was employed as a multidentate growth-mediating ligand, efficiently passivating the NC surface. Briefly, PbBr_2_, MABr, and MLA were first solubilized in dimethylformamide to form a stock solution. Subsequently, oleylamine (OLAm) was added, yielding a white suspension, which was then added dropwise into toluene, serving as a nonsolvent solvent for the reaction (Figure 1a). The resulting mixture was stirred for 2–4 h, yielding a pale-green colloid that exhibited bright photoluminescence (PL) (Figure 1b). The progress of the MAPbBr_3_ NPL synthesis was continuously monitored with in situ optical absorption spectroscopy (Figure S1). Over the first ca. 10 min, laterally confined 2-monolayer (ML)-thick MAPbBr_3_ NPLs formed, exhibiting their first excitonic transition at approximately 427 nm (Video S1). We note that this energy is slightly higher than that of 431 nm for laterally extended 2 ML MAPbBr_3_ NPLs.^12^ The second and third excitonic transitions—characteristic of LHP NPLs^67,68^ —also emerged in the absorption profile. At this early stage of reaction progress, the observation of broadened absorption features corroborates the coexistence of multiple species. Over the course of several hours, the primary absorption peak gradually red-shifted, eventually reaching wavelengths of 445–450 nm, typical for MAPbBr_3_ NPLs with a thickness of 3 MLs (see also Video S2).^69^ Notably, no abrupt shifts were observed in the absorption spectra at this stage, indicating the absence of temporally correlated quasi-instantaneous NPL thickening across the NPL ensemble. Therefore, we hypothesize simultaneous growth of NPLs in lateral and thickness directions, with both 2 and 3 ML NPLs present in the intermediate stage of the reaction.

**Figure 1:**
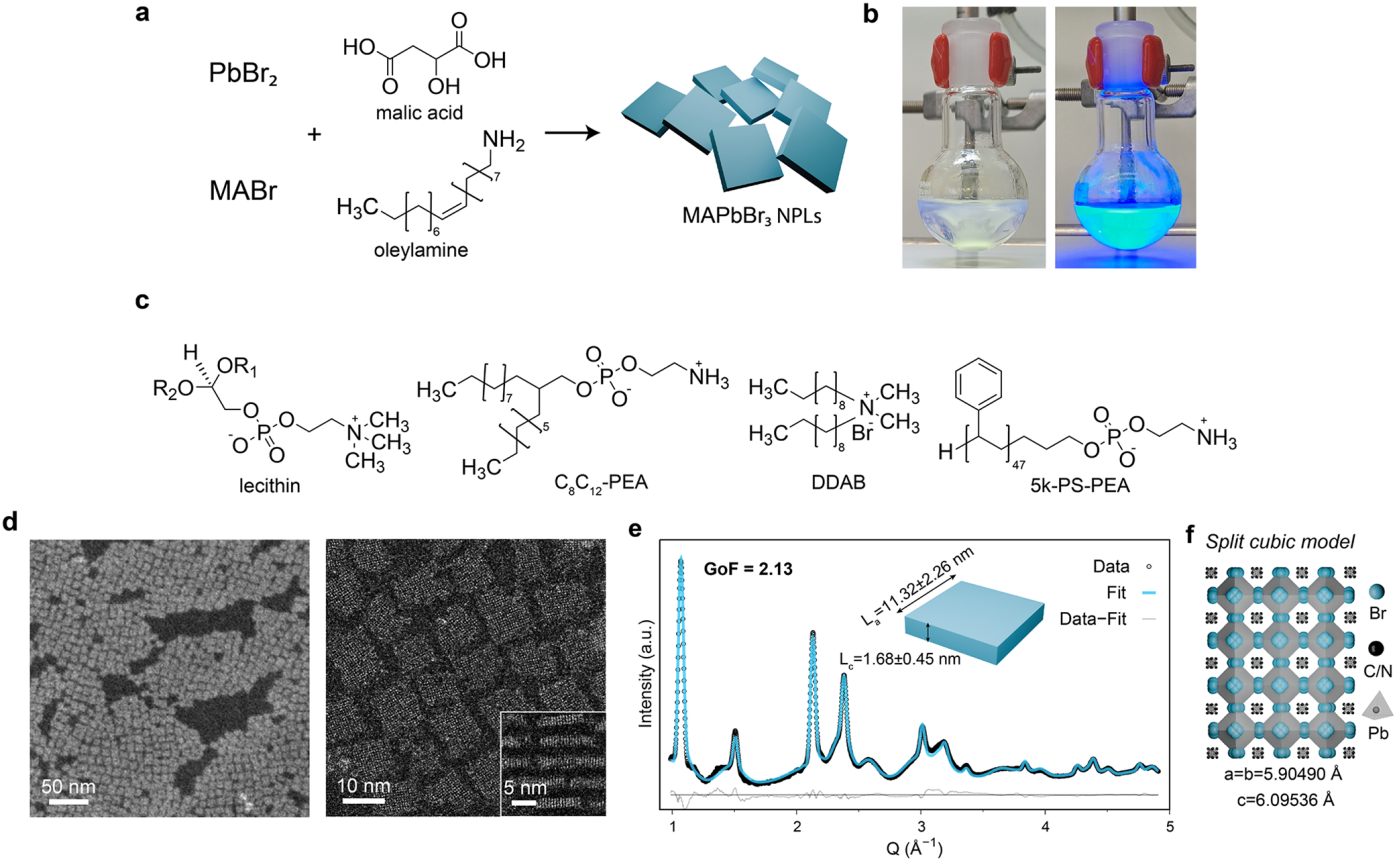
Synthesis of MAPbBr_3_ NPLs. (a) Reaction scheme for the synthesis of MAPbBr_3_ NPLs. (b) The reaction mixture at the end of the synthesis under ambient light (left) and under UV irradiation (right), revealing the bright blue PL of the synthesized NPLs. (c) Organic ligands used for the postsynthetic treatment of pristine MAPbBr_3_ NPLs, capped with OLAm and MLA. (d) DF-STEM (left) and HAADF-STEM (right) images of lecithin-capped MAPbBr_3_ NPL monolayer, demonstrating a high degree of monodispersity in lateral dimensions, with (inset) HAADF-STEM image of vertically oriented NPLs. (e) The Debye scattering equation (DSE) best fit of the solvent-subtracted colloidal C_8_C_12_–PEA-capped MAPbBr_3_ NPLs data, obtained using the tetragonally distorted structural model. (f) The split model of the tetragonally distorted MAPbBr_3_ NPL structure, where the disorder of Br^–^ anions around their “ideal” positions is modeled by splitting each one into four equivalent sites with 1/4 occupancy. On average, the Pb–Br–Pb angle is 164.5° (details in the Methods).

As-synthesized MAPbBr_3_ NPLs are initially capped with OLAm and MLA ligands. Owing to the dynamic binding of OLAm,^57^ the NPLs do not exhibit sufficient stability toward dilution, purification with antisolvent, or long-term storage. These stability issues hinder structural and optical characterization of the NPLs, necessitating attempts to conduct single-dot spectroscopy after substantial dilution. To overcome these challenges, ligand exchange with more potent capping ligands was carried out (Figure 1c): lecithin (natural phosphocholine),^62^ didodecyldimethylammonium bromide (DDAB),^70,71^ and phosphoethanolamine­(PEA)-based ligands,^61^ namely C_8_C_12_–PEA and 5k-polystyrene­(PS)-PEA. The treated NPLs retained bright PL, colloidal stability, and structural integrity, as evidenced by transmission electron microscopy (TEM) imaging (Figure S2). In contrast to NPLs capped with pristine ligands, the ligand-exchanged NPLs can be purified multiple times without losing colloidal stability, maintaining shape and size uniformity (Figure S3). This enhanced stability across multiple washing steps can be partially attributed to the use of the antisolvent dimethyl carbonate (DMC).^66^ We then selected two zwitterionic ligands, lecithin and C_8_C_12_–PEA, which performed best for postsynthetic ligand exchange. MAPbBr_3_ NPLs capped with either of these ligands exhibited high PLQY values of 60–80% in a colloidal solution, highlighting the importance of zwitterionic ligands for surface passivation. Notably, lecithin- and C_8_C_12_–PEA-capped NPLs demonstrated long-term environmental stability, showing only slight redshifts (ca. 2–3 nm) in absorption and emission spectra after 2 months of air exposure (Figure S4). Compared to lecithin-capped MAPbBr_3_ NPLs, C_8_C_12_–PEA-capped NPLs preserved PLQY more effectively over 2 months’ storage and showed no signs of degradation based on TEM imaging. Overall, such uncommon stability of strongly confined LHP NCs can be attributed to two primary factors: (i) the zwitterionic headgroup of the ligand stabilizing the NPL surface^61,62,72^ and (ii) the formation of stacked face-to-face assemblies within the NPL colloid can provide additional collective stability.^2,5^

Structurally, synthesized MAPbBr_3_ NPLs exhibit a uniform square shape in lateral dimensions, with a typical edge length of 10–15 nm (Figure 1d). High-angle annular dark field scanning transmission electron microscopy (HAADF-STEM) imaging of the MAPbBr_3_ NPLs (Figure 1d inset) revealed 3 ML-thick NPLs (1.8 nm in thickness), exhibiting a high degree of monodispersity along the [001] direction. These findings are in line with the wide-angle X-ray total scattering (WAXTS) analysis of synchrotron data collected on NPL colloidal solutions (Figure 1e), which yield the refined number-based sizes of L_a_ = 11.32 nm (σ/L_a_ = 20%) and L_c_ = 1.68 nm (σ/L_c_ = 27%), consistent with 3 ML-thick NPLs with a minor fraction of 2 ML NPLs. Notably, this analysis demonstrated a tetragonal distortion (*P4/mmm*) of the bulk MAPbBr_3_ cubic structure, driven by strong NPL quantum confinement resulting in unit cell expansion along the NPL thickness direction (*c*/*a* > 1). The refined unit cell parameters were determined to be a = b = 5.905(1) Å and c = 6.095(3) Å (Figure 1f, details in the Methods). A model with a cubic structure (refined unit cell a = 5.903(1) Å; Figure S5) yielded a poorer fit than the tetragonal model. The observed tetragonal distortion of the cubic unit cell (*P4/mmm*) is size-induced, as it was not present in cubic-shaped MAPbBr_3_ NCs, typically displaying a cubic structure with a = 5.92149(7) Å or in bulk MAPbBr_3_ (cubic structure with a unit cell parameter of a = 5.93076(2) Å).^73^

The systematic variation in the A-cation and halide compositions of LHP NPLs provides a robust handle not only for spectral tunability but also offers a pathway to tailor their bandgap and exciton energies.^12,74^ We therefore attempted to synthesize mixed-halide MAPb­(Br/Cl)_3_ NPLs by introducing MACl into the precursor solution (Figure S6). The absorption and PL spectra of MAPb­(Br/Cl)_3_ NPLs are blue-shifted by up to 25 nm while preserving their narrow spectral features. In addition, the PLQY values remain as high as 60%, even in the NPLs with 25% Cl content. TEM characterization confirmed the structural integrity of MAPb­(Br/Cl)_3_ NPLs with up to 38% Cl content. However, at higher Cl content, the NPLs became unstable in the crude solution, rapidly dissolving as the reaction progressed. Another avenue for exploring the compositional space is to replace MA^+^ with another typical A-cation, *e.g.,* FA^+^ or Cs^+^. Very rapid growth kinetics led to the formation of nonemissive bulk FAPbBr_3_, rather than NPLs, via an analogous synthesis. Monodisperse CsPbBr_3_ NPLs with lateral dimensions of 19.5 ± 2.7 nm and a thickness of 1.7 ± 0.2 nm, however, were successfully synthesized and purified (Figure S7).

### Assemblies of MAPbBr_3_ NPLs

Semiconductor NPLs are inherently prone to self-assemble into highly ordered structures, largely due to the attractive van der Waals forces acting between their basal planes.^2,4,5,75–78^ MAPbBr_3_ NPLs form stacked face-to-face assemblies already upon drying (Figure 2a). The typical domain sizes amounted to 1–2 μm on average. Notably, the periodicity of stacked NPLs in the assembly was determined to be 4.8 nm by HAADF-STEM imaging (Figure 2b), which accounts for the intrinsic NPL thickness of 1.8 nm, corresponding to 3 ML, and two ligand shells, 1.5 nm each.^1,2,48^ The drop-cast films were analyzed using a powder diffractometer in transmission mode (Figures 2c and 2d). The wide-angle XRD pattern (Figure 2c) displays peaks attributed to the cubic MAPbBr_3_ phase. At lower angles (Figure 2d), SL peaks (n = 2–4, 6–9) corresponding to NPL stacking are determined by the occurrence of harmonic peaks of the fundamental one, falling at q = 0.128 Å^–1^, or, equivalently, at d = 2π/q = 49 Å, in excellent agreement with the value observed with HAADF-STEM characterization. Synchrotron-based grazing-incidence wide-angle X-ray scattering (GIWAXS, Figure 2e and f) measurement was performed on MAPbBr_3_ NPL assemblies deposited onto optically transparent silicon nitride (SiN_*x*_) membranes. The low-angle region of the GIWAXS pattern shows rings corresponding to NPL ordering on a SiN_*x*_ membrane, again with a periodicity of approximately 4.9 nm. An azimuthal-integrated 1D pattern in the WAXS region (Figure 2g) revealed the Bragg peaks for the MAPbBr_3_ phase along with the SL peaks. Notably, the low-angle region of the collected WAXS data set (Figure S8) for NPL colloids contained SL reflections across all tested MAPbBr_3_ NPLs capped with OLAm, C_8_C_12_–PEA, or lecithin, indicating the presence of NPL assemblies in their colloidal solutions, though at a slightly expanded stacking periodicity (up to 5.4 nm).

**Figure 2:**
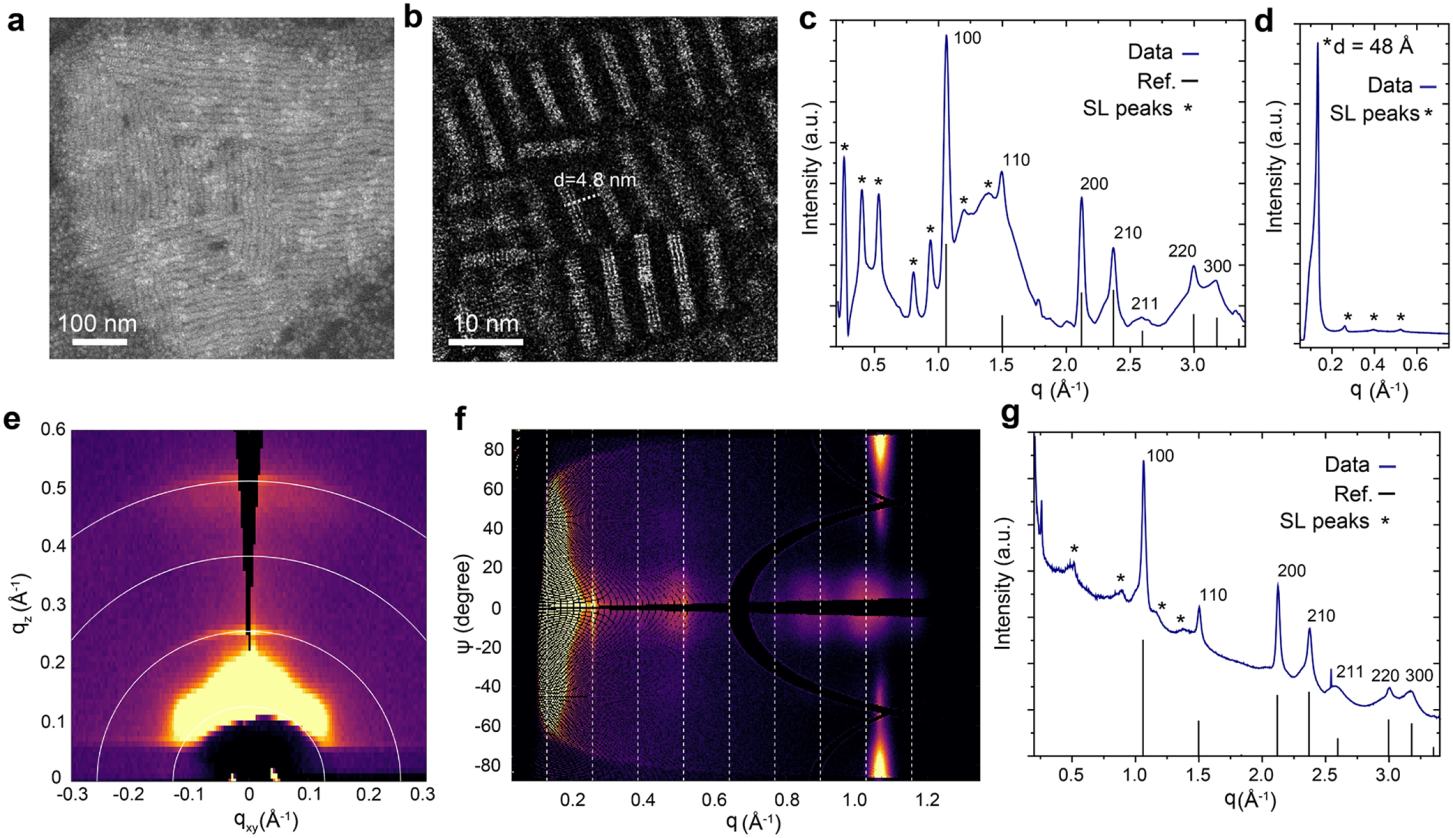
Assemblies of MAPbBr_3_ NPLs. (a) DF-STEM and (b) HAADF-STEM images of lecithin-capped MAPbBr_3_ NPL assemblies demonstrating sub-μm domain dimensions and face-to-face NPL stacking, respectively. (c) High-angle region of the XRD pattern of MAPbBr_3_ NPL drop-cast film (dark blue line) displaying peaks at *q* > 1.0 Å^–1^, attributed to the MAPbBr_3_ phase, and the diffraction peaks corresponding to the NPL stacking (q = 2πn/d with d = 49 Å and *n* = 2–4, 6–9, marked with an asterisk). The *n* = 5 peak is absent as it is structurally too weak to be detected (Figure S8). Black vertical lines denote reference reflections for single-crystal MAPbBr_3_ (*Pm3̅m*, ICSD 252415). (d) Low-angle region of the XRD pattern of MAPbBr_3_ NPL drop-cast film (dark blue line), revealing the first peak (*n* = 1, marked with an asterisk) corresponding to the NPL periodicity of 4.8 nm, depicted in (b). (e) 2D low-angle region of GIWAXS pattern showing the rings corresponding to the NPL periodicity. (f) The polar plot of the 2D GIWAXS pattern in the *q* < 1.4 Å^–1^ region. The rings corresponding to the periodic arrangement of NPL are transformed into vertical lines in the polar plot; the *n* = 5 reflection is absent. The ψ = ± 90° peaks at q = 1.06 Å^–1^ belong to the MAPbBr_3_ phase (100 reflection in the cubic notation). (g) Azimuthally integrated 1D pattern in the *q* > 0.2 Å^–1^ region (dark blue line), revealing Bragg peaks for the MAPbBr_3_ phase (black vertical lines) and the diffraction peaks corresponding to NPL stacking (marked with an asterisk).

### NMR Studies on MAPbBr_3_ NPLs

To assess the ligand exchange from primary ammonium to zwitterionic ligands, ^1^H NMR spectra were collected from MAPbBr_3_ NPLs treated with increasing concentrations of the zwitterionic ligands. The spectra of MAPbBr_3_ NPLs treated with C_8_C_12_–PEA consistently showed a decrease in several signals assigned to OLAm at 5.7, 4.1, and 3.7 ppm (Figure 3a, marked with an asterisk), while the signal at 3.5 ppm associated with the MA in the NPLs remained unchanged. Similarly, NPLs treated with lecithin exhibit decreased peak intensities associated with OLAm. This provides clear evidence that OLAm is removed by the ligand treatment while the structure of the NPLs remains intact. We note that the line widths of the peaks associated with OLAm suggest an unprecedentedly tight binding of the ligand to the NPLs, likely due to NPL stacking in solution, even at concentrations as low as 1 mg/mL. Notably, the second growth-mediating ligand employed in the synthesis, MLA, was not observed in the NMR spectrum, as it is either washed out during purification with an antisolvent or entirely replaced during ligand exchange.

**Figure 3:**
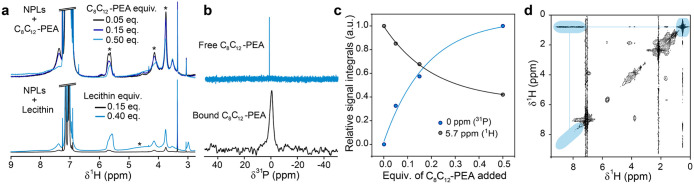
NMR studies on MAPbBr_3_ NPLs. (a) ^1^H NMR spectra of MAPbBr_3_ NPL colloidal solutions treated with different amounts of C_8_C_12_–PEA (0.05, 0.15, and 0.5 equiv. with respect to initial OLAm) and lecithin (0.15 and 0.4 equiv. with respect to OLAm), demonstrating reduction of OLAm signal intensity upon higher amounts of added ligand. (b) ^31^P NMR spectra of C_8_C_12_–PEA-capped MAPbBr_3_ NPLs in toluene-d_8_ (black) and digested in DMSO-*d*_6_ (blue). The broadened feature in the former spectrum, as opposed to the spectrum of digested NPLs, signifies the bound nature of the zwitterionic ligand to the surface. (c) The dependence of ^1^H and ^31^P signals on the amount of C_8_C_12_–PEA added revealing the partial replacement of OLAm. (d) NOESY spectrum of C_8_C_12_–PEA-capped MAPbBr_3_ NPLs in toluene-d_8_ demonstrating the correlation between −NH_3_^+^ and −CH_3_ protons (marked with blue).

To gain insight into the added ligands, ^31^P NMR spectra were recorded, leveraging the phosphorus atom present in C_8_C_12_–PEA and lecithin. A single broad feature associated with the bound zwitterionic ligands was observed (Figure 3b), scaling with the amount of ligand added during the treatment. To confirm the bound nature of the ligands, the NPLs were decomposed with DMSO-*d*_6_, and the sample was measured to reveal a single narrow signal associated with C_8_C_12_–PEA. With both data sets available, we compared the decrease in the ^1^H peaks of OLAm with the corresponding increase in the ^31^P signature of the zwitterionic ligands (Figure 3c). Both parameters exhibited strong correlation across the tested range of treatment concentrations following an exponential function. Overall, we can confirm the successful partial ligand exchange from OLAm to either C_8_C_12_–PEA or lecithin.

In addition, we conducted a TEM study on NPLs treated with different amounts of ligands (Figure S9). Notably, the NPLs stayed intact at 0.03, 0.18, and 0.8 equiv of added C_8_C_12_–PEA with respect to OLAm, whereas NPL merging and degradation were observable at 1.6 equiv of the zwitterionic ligand. This suggests the necessity of OLAm presence on the surface for NPL integrity in a colloidal solution.

Finally, to address the stacking between neighboring NPLs, a nuclear Overhauser effect spectrum (NOESY) was recorded with a short mixing time (50 ms). Apart from the expected correlation peaks between neighboring protons, we also observed a strong correlation between the -R-NH_3_^+^ and the −CH_3_ protons, both before and after the ligand exchange (Figure 3d). Such a correlation between opposite ends of the ligands can only arise if a significant number of NPLs are tightly stacked, e.g., when the ends of the ligands of an NPL are close to the surfaces of neighboring NPLs. Although this frequently occurs in NC SLs, we observed NPL stacking in solution, even at low concentrations, in solvents native to the ligands’ tails. Such stacking behavior explains the enhanced stability of MAPbBr_3_ NPLs against prolonged air exposure and repeated washings.

### Optical Properties of MAPbBr_3_ NPL Ensemble

Next, we investigated the luminescence of synthesized MAPbBr_3_ NPLs to explore the underlying excitonic photophysics. At cryogenic temperatures, reduced thermal fluctuation narrows the spectral features, revealing the fundamental properties of the material. For example, the PL spectrum of a densely packed NPL film at 4 K exhibited (i) a sharp excitonic emission at a similar energy to that at RT and (ii) a broader, less emissive feature at lower energy (Figure 4a). Such multicolor emission at low temperatures resembles that widely observed for CdSe NPLs, the origin of which is attributed to the mixed contributions of trion and excimer emission.^25,79,80^ MAPbBr_3_ NPLs here exhibited the transient photodarkening of trion emission at a similar time scale with that in CdSe NPL (several seconds; Figure S10) but with a larger binding energy (∼200 meV red-shifted from the excitonic emission compared to ∼28 meV in 4 ML CdSe NPL),^80^ likely owing to a thinner NPL thickness and a more ionic character of the organic–inorganic perovskite lattice. At lower concentrations of NPLs, such low-energy features reduced and completely vanished for isolated NPL clusters, which only exhibited sharp excitonic emission (Figure 4a), excluding the potential contributions of self-trapped or defect-bound excitons to the red-shifted band observed in NPL assemblies. This concentration dependence clearly reveals the NPL-NPL stacking effect and thus the contribution of excimeric/trion emission from electronically coupled NPL dimers. Indeed, the slow PL decay probed at 2.5 eV exhibits the characteristic decelerated emission of an excimer, in contrast to the faster excitonic emission decay probed at 2.7 eV (Figure 4a, inset). At elevated temperatures, these low-energy features in dense MAPbBr_3_ NPL ensemble disappeared, likely due to the Auger-quenched trion and/or thermally perturbed NPL-NPL excited-state coupling (Figure 4b, S10). Although commonly observed in CdSe NPLs, such multicolor emission appears to be exclusive to MA-counterparts among LHP NPLs and absent in CsPbBr_3_ (Figure S11) or FAPbBr_3_ NPLs.^81^ Such behavior is likely associated with the differences in surface properties and assembly tendencies that vary with compositions.

**Figure 4:**
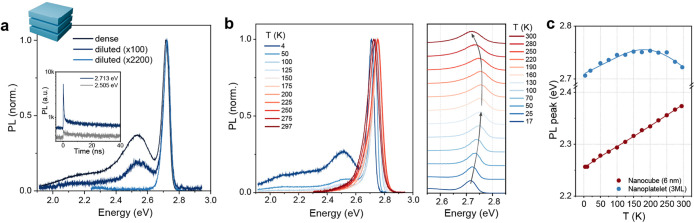
Optical properties of MAPbBr_3_ NPL ensemble. (a) Normalized PL spectra at 4 K of lecithin-capped MAPbBr_3_ NPL films with varying concentrations. Dense: 2 mg/mL solution drop-cast; diluted: 0.02 mg/mL solution drop-cast; diluted: 0.0009 mg/mL solution spin-coated. (inset) PL decays of PL peaks at 2.713 eV (457 nm, blue) and 2.505 eV (495 nm, gray) for dense MAPbBr_3_ NPL film. (b) Left: normalized PL spectra of dense C_8_C_12_–PEA-capped MAPbBr_3_ NPL film at varying temperatures, 4–297 K. Right: magnified excitonic emission peak showing nonmonotonous peak energy evolution across temperatures. (c) Temperature evolution of PL peak energy of MAPbBr_3_ NPLs (3 ML thick, blue) and of MAPbBr_3_ cubic NCs with a comparable volume (6 nm edge length, red). Solid lines are the fits with the Bose−Einstein single-oscillator model.

Temperature dependence of NPL excitonic emission reveals the intriguing nature of its quantum confinement and exciton–phonon interactions. For most covalent semiconductors, it is generally observed that the bandgap energy blueshifts with decreasing temperature due to thermal contraction and phonon suppression, following Varshni’s empirical relation. However, for LHP, the opposite is known to be universal: bandgap redshifts at lower temperatures with lattice contraction.^82–85^ Indeed, this was observed for MAPbBr_3_ cubic NCs (edge length 6 nm, similar volume with NPL), which showed a monotonous redshift from RT to 4 K (Figure 4c, Figure S12). However, MAPbBr_3_ NPLs exhibited clearly distinct behavior, showing a nonmonotonous peak shift across the temperature (Figure 4b and c). Upon cooling from RT, its PL peak exhibited an initial blueshift until approximately 200 K, after which it red-shifted.

Such an unconventional temperature dependence of PL points to strong anisotropic confinement of the exciton in atomically thin NPL. 2D layered perovskites were reported to show a transition from the (perovskite) conventional redshift to a blue-redshift turnover, then eventually to blueshift as the layer thickness decreased from n = 5 to n = 1.^86^ CsPbBr_3_ NPLs (<3 ML) also exhibited nonmonotonic evolution of *E*_g_(*T*).^78,87,88^ In CsPbBr_3_ NPL, such a crossover is attributed to changes in the phonon structure arising from the two-dimensional nature of quantum confinement, particularly the increased contribution of optical phonons at the expense of acoustic phonons.^88^ Although a similar anisotropy-driven change in phonon texture and the resulting PL crossover are also observed in the MAPbBr_3_ NPLs studied here, the underlying origin may be more complex—an interplay of phase transitions across the temperature range and associated changes in the thermal expansion coefficients and phonon modes. When the capping ligand was varied from C_8_C_12_–PEA to lecithin, MAPbBr_3_ NPLs still exhibited PL crossover with temperature (). In addition, we note that CsPbBr_3_ NPLs (4 ML) characterized in this study () exhibited a monotonous temperature-dependent progression of the PL peak, as reported previously.^87^

### Optical Properties of Single MAPbBr_3_ NPL

Fundamental photophysics of NPL excitons can be revealed by removing the ensemble averaging effect when characterizing individual MAPbBr_3_ NPLs. However, the high propensity of NPLs to stack (Figure 2a) presented a challenge to probe the signal from an isolated single NPL. To overcome this problem, lecithin-capped NPLs were employed as they exhibited a lower tendency to self-assembly compared to both C_8_C_12_–PEA-and OLAm-capped NPLs (Figure S2 and S4). Furthermore, a highly diluted NPL solution was embedded in a polymer matrix to further circumvent NPL stacking. Despite the efforts, PL signals from isolated clusters of stacked NPLs were still often observed, one example of which is shown in Figure 4a, upon high dilution. Nevertheless, through careful screening, we successfully identified and characterized individual, isolated NPLs, which revealed intriguing photophysical properties.

At RT, single NPL emission exhibited a rather asymmetric PL peak with a line width (full width at half-maximum, fwhm) of 90 meV (Figure 5a). At cryogenic temperature (4 K), single NPL emission narrowed with PL line width lower than 20 meV (Figure 5b and c), which is much narrower than that of the cluster emission shown in Figure 4a. Such line-narrowing at lower temperatures attests to the dominance of exciton–phonon coupling on single NPL emission at higher temperatures. However, the magnitude of line-narrowing was not as dramatic as in single cubic MAPbBr_3_ NCs, where the zero-phonon line (ZPL) narrowed down to ∼1 meV (Figure S14). This reflects the strong confinement exerted on the NPL exciton, of which the extensive interaction with surface atoms makes it more prone to spectral broadening. Indeed, time-resolved PL decay reveals a radiative lifetime of 1.8 ns (Figure 5d), which is relatively long for a perovskite NC at cryogenic temperature.^84,89^ This lack of e-h correlation and thus the lack of single-photon superradiance compared to the cubic-shaped LHP NCs with similar lateral dimensions,^84^ is another indication of strong exciton anisotropic confinement in MAPbBr_3_ NPLs.

**Figure 5:**
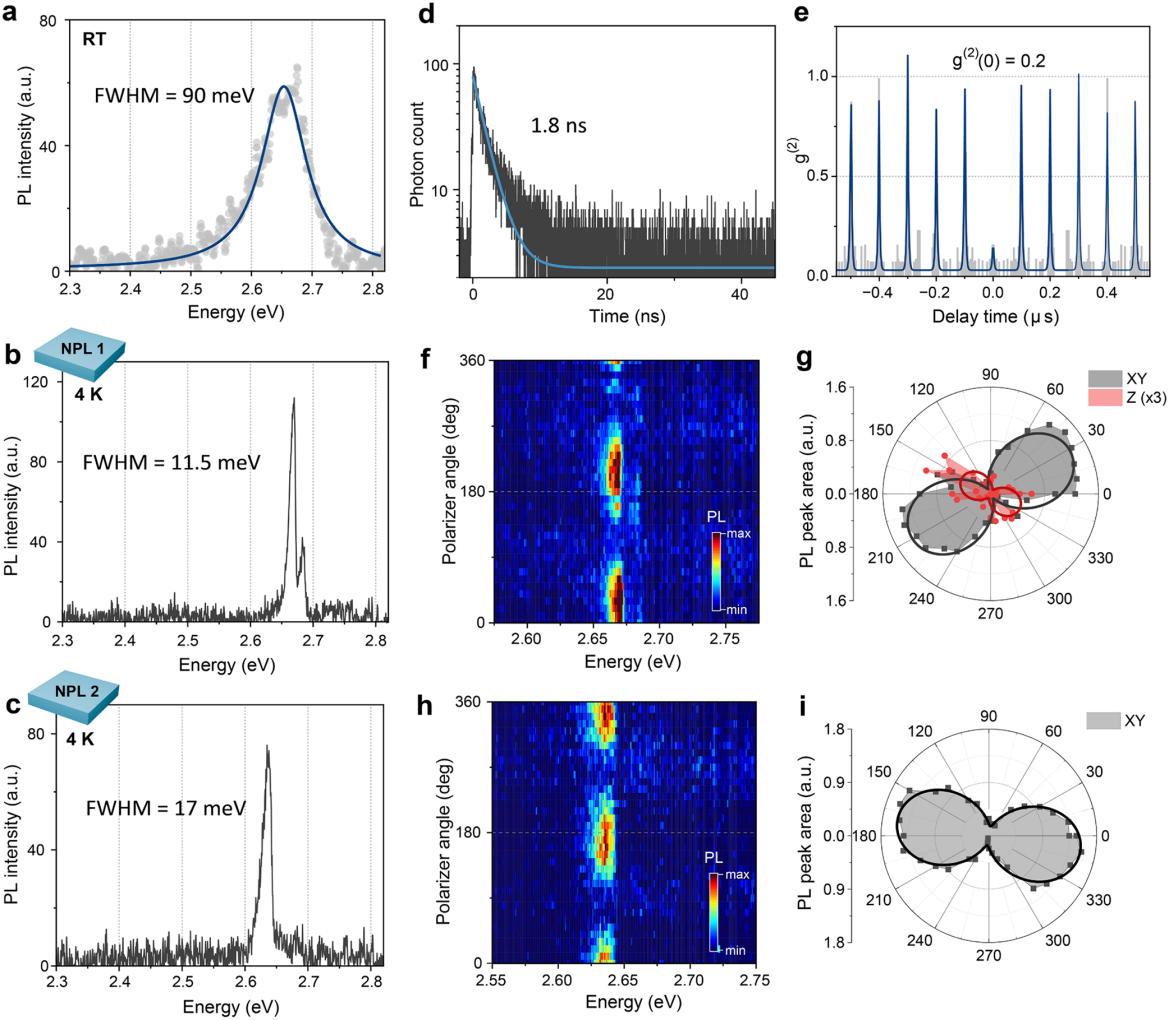
PL properties of single MAPbBr_3_ NPL. (a) PL spectrum of a single lecithin-capped MAPbBr_3_ NPL at room temperature. The blue solid line is the fit with the Lorentzian function. (b,c) PL spectra of single NPLs—NPL1 and NPL2, respectively—at cryogenic temperature of 4 K. PL line widths were 11 and 5 meV for the lower and higher energy peaks of NPL1 and 17 meV for NPL2. (d) Time-resolved PL decay trace of NPL2. The blue line is the fit with the monoexponential decay function. (e) Second-order intensity correlation of NPL2 showing a highly antibunched photon statistics. (f,h) Polarization-resolved PL spectra series for corresponding single NPLs at 4 K. (g,i) Polar plots of observed PL peaks for corresponding single NPLs at 4 K.

Second-order correlation measurements (g^(2)^) obtained from single NPL emission via the Hanbury-Brown-Twiss setup showed pronounced antibunching, confirming the quantum nature of NPL emission (Figure 5e). Considering that 2D layered perovskites, the closest electronic analogue to colloidal NPLs, do not emit single photons, MAPbBr_3_ NPLs stand out as a versatile candidate for quantum light sources, not only by being atomically defined in thickness but also by being laterally confined and behaving as a quantum emitter.

Corresponding single NPL emissions were subjected to polarization-resolved measurements, which revealed highly linearly polarized emission dipoles (Figure 5f−i). As shown in the gray area and black fit curves in the polar plot, the emission peaks were characterized by degrees of polarization (DOP) of 88% and 81%, respectively, for NPL1 and NPL2 (Figure 5g,i). We attribute these emission lines to the in-plane (X and Y) dipoles of NPL, which are expected to be the lowest emitting state, with the X and Y dipoles being nearly degenerate.^90^ The orientation of emission dipoles is determined by the relative configuration of the NPLs with respect to the optical axis. In this context, one can visualize either the X or Y dipole, aligning more closely with the optical axis, making its emission dipole less likely to be optically resolved. In contrast, the orthogonal dipole, being more perpendicular to the optical axis, exhibits a geometry that is highly sensitive to polarization, making it more easily resolvable. This anisotropic alignment accounts for the observed nonunity degree of polarization (DOP) in NPL emissions. Interestingly, a sharp emission line was observed at 12.9 meV above the main emission peak for NPL1 (Figure 5b). Polarization-resolved results attest that this emission dipole was oriented nearly orthogonal to the main emission peak (Figure 5f and g). Therefore, this higher-energy emission line is likely the fine-structure state of the NPL exciton oriented out of plane (Z). We attribute the lifted degeneracy of this state to the anisotropic confinement on par with the structural elongation of a unit cell along the out-of-plane direction (Figure 1f). Moreover, the XY-Z splitting energy observed here matches well with the theoretically predicted value, which suggests a 10–12 meV XY-Z splitting energy for 3 ML thick NPL.^90^

### Exciton-Polariton Formation in MAPbBr_3_ NPL Assemblies

The potential to control and enhance the optoelectronic properties of LHPs through interaction with optical resonances has motivated numerous attempts to integrate them into photonic structures. Much efforts have been focused on reaching the so-called strong light-matter coupling regime, in which the exchange of energy between excitons and cavity modes occurs at shorter times than the lifetime of the excited electron state and the dephasing time of photons.^91^ Under these premises, the optical response of the system is no longer determined by the individual exciton and photon states but by hybrid modes known as exciton-polaritons. The opportunities that polaritons offer to shape light absorption, emission, and propagation have been leveraged to develop a wide range of perovskite-based optical devices with enhanced performance.^92–99^

A necessary condition for polariton formation is the spectral overlap of a highly localized optical resonance and a high-oscillator-strength excitonic transition—intrinsic attributes of LHP NPLs. We therefore endeavored to explore the formation of NPL films of different thicknesses by means of drop-casting and spin-coating. The deposition of NPL octane/hexane colloids (1:2 v/v) on a glass substrate yielded optically transparent and uniform films with preserved bright PL emission (Figure 6a inset). Analysis of film absorption spectra revealed the absence of background absorption and, hence, nonscattering of light by both thick drop-cast and thin spin-coated films (Figure 6a and S15). Notably, sharp excitonic features were preserved in film absorption, as evident from superimposed spectra of a film and the respective colloidal solution. Atomic force microscopy (AFM) revealed a high degree of film uniformity, with a root-mean-square (RMS) roughness of 4.2 nm and a thickness of approximately 200 nm for a drop-cast film (Figure 6b and c).

**Figure 6:**
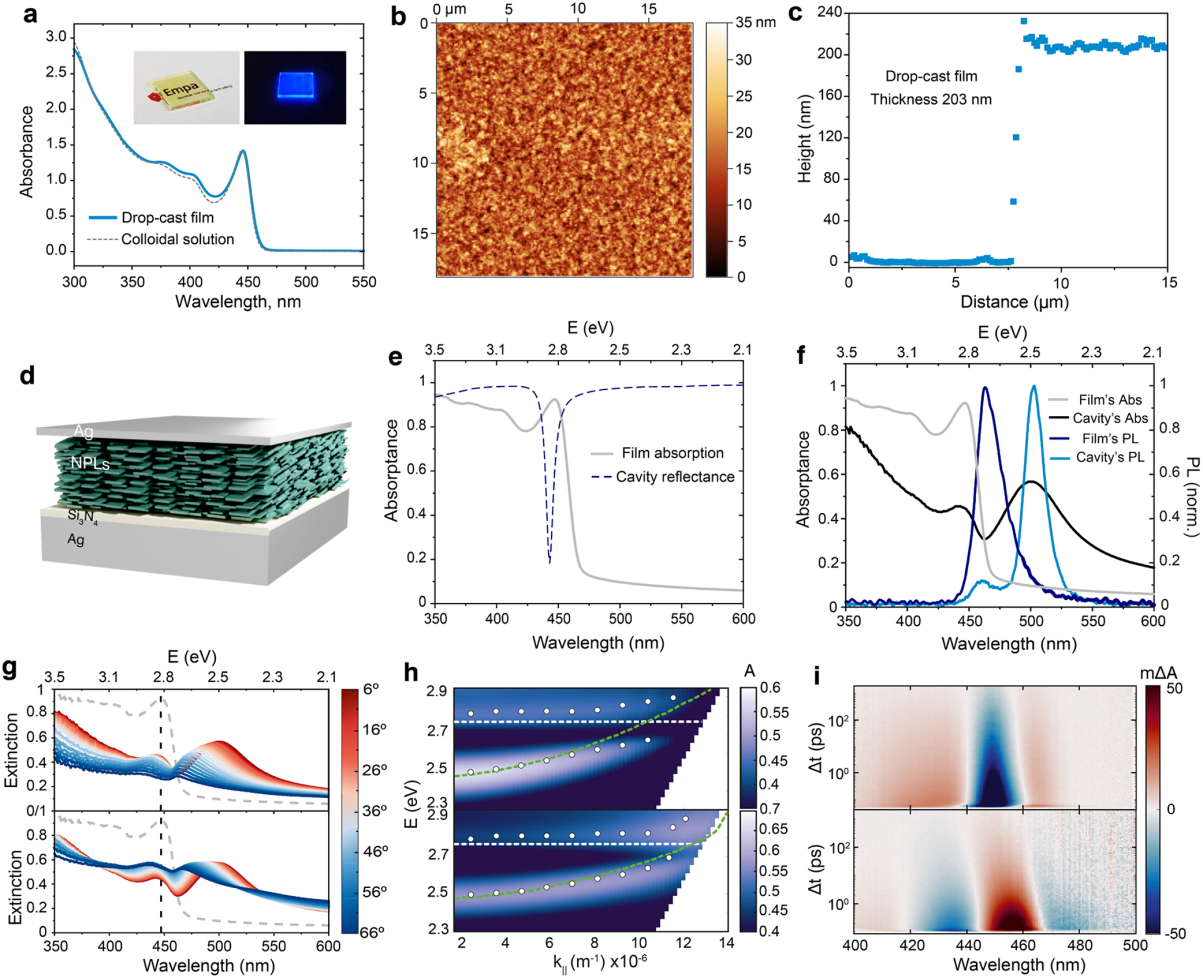
Exciton-polariton formation in MAPbBr_3_ NPL assemblies. (a) Absorption spectra of the drop-cast film and corresponding colloidal solution of lecithin-capped MAPbBr_3_ NPLs are depicted with blue and gray dashed lines, respectively. The absence of a background intensity in the absorption spectra of either film demonstrates negligible light scattering and hence a high degree of uniformity and smoothness of the fabricated films. (inset) Appearance of drop-cast film of lecithin-capped MAPbBr_3_ NPLs under ambient light (left) and UV excitation (right), demonstrating film transparency and bright blue PL. The logo is reproduced with permission from the Swiss Federal Laboratory for Materials Science and Technology (Empa, Switzerland). (b) AFM image of drop-cast film with an RMS roughness of 4.2 nm. (c) Cross-sectional height profile of drop-cast film with a thickness of ca. 203 nm. (d) Illustrative drawing of an NPL assembly film embedded in an optical cavity. (e) Absorptance of the NPL film (light gray solid line, experimental) and reflectance of the underlying cavity mode (dark blue dashed line, calculated assuming the actual parameters employed to build the resonator). (f) Absorptance of the NPL optical cavity (black solid line) and photoemission of the bare NPL film (dark blue solid line) and the NPL cavity (blue solid line); the NPL absorptance is plotted for the sake of comparison. (g) Angle-dependent absorptance for TE (upper panel) and TM (lower panel) polarized incident light. (h) Absorptance energy dispersion maps for TE (upper panel) and TM (lower panel) polarizations of the incident beam; white dots indicate the spectral position of the experimental absorption maxima, horizontal white dotted lines pinpoint the positions of the 1s-1s excitonic transition, and green dotted lines represent the underlying cavity mode dispersion. (i) Maps of ΔA versus λ and Δt attained from ultrafast transition absorption spectroscopy measurements for the NPL film (upper panel) and NPL optical cavity (lower panel).

The high quality of fabricated films makes MAPbBr_3_ NPLs suitable candidates for integration into Fabry-Pérot resonators to explore strong light-matter coupling. With this idea in mind, we deposited a 250 nm thick NPL film onto a SiN_*x*_ (15 nm) protected Ag bottom mirror (200 nm) sputtered on glass. Then, a thin top Ag mirror (30 nm) was thermally evaporated onto the upper surface of the NPL film to close the resonator; see a drawing of one of these optical cavities in Figure 6d. In this example, we used slabs made of lecithin-capped NPLs, as they exhibited greater homogeneity and flatness than C_8_C_12_–PEA-capped NPLs. This specific design ensures the intersection between the resonant cavity dispersion, in particular, that corresponding to the second-order mode, and the spectral excitonic transition of the NPLs, as shown in Figure 6e. The underlying cavity response was simulated using a transfer-matrix method (TMM) code, with the optical constants of all materials in the resonator as input, as provided in Figure S16. The dashed line in Figure 6e corresponds to the reflectance of TE (transversal electric) polarized light incident at an angle of 50° with respect to the surface normal. The effect of the strong light-matter coupling could be readily seen in the absorptance of the cavity (black solid line in Figure 6f), which displays two peaks corresponding to the transitions from the ground state to the lower and upper polaritons, located at λ ≈ 500 nm and λ ≈ 445 nm, respectively. Polariton emission (blue solid line, λ_max_ ≈ 500 nm) is channeled through the lower polariton and shows a significant 210 meV redshift with respect to the luminescence resulting from the radiative recombination of excitons in the bare NPL ensemble (dark blue solid line, λ_max_ ≈ 460 nm).

Conclusive evidence of polariton formation is provided by the analysis of the angle-dependent absorptance, shown in Figure 6g for both TE (upper panel) and TM (transversal magnetic, lower panel) polarized light. The energy dispersion absorption maps attained by plotting these measurements versus the photon energy (*y*-axis) and the parallel component of the incident light wavevector **k**∥ (*x*-axis, **k** = **k**∥ + **k**⊥) for the two polarizations in Figure 6h (TE, upper panel; TM, lower panel), reveal the avoided crossing of the two polaritonic branches, which constitutes the definitive signature of strong light-matter coupling. In fact, a significant Rabi splitting of ℏΩ ≈ 200 meV can be estimated from these graphs at the intersection of the cavity mode dispersion curve (green dashed line) and excitonic transition (white dashed line). Notably, this value is significantly larger than those so far achieved in LHP NCs^64,65^ and equal to the largest achieved for low-dimensional 2D or quasi-2D perovskite phases strongly coupled to photon resonances.^100,101^ For comparison, simulated absorption dispersion maps calculated using the TMM are plotted alongside the experimental data for both polarizations in Figure S17, showing reasonable agreement. Figure S18 shows the calculated spatial and spectral distributions of the electric-field intensity and absorption for different incident-light angles, which allow visualization of mode splitting, characteristic of strong light-matter coupling, and confirm that polariton absorption occurs mainly in the NPL ensemble.

Finally, exciton and polariton excitation and decay dynamics in both NPL films and optical cavities were studied and compared by ultrafast transient absorption spectroscopy (TAS). Results are presented as intensity maps in Figure 6i, where the full series of ΔA spectra, attained by subtracting the absorbance of the nonexcited film (upper panel) or cavity (lower panel) from that of the photoexcited one, are plotted as a function of probe photon wavelength (*x*-axis) and pump–probe delay time (*y*-axis), *Δt*. Excitation wavelengths employed were λ = 400 nm for the film, above the excitonic transition, and λ = 440 nm for the cavity, in resonance with the upper polariton. The fluence of the 190 fs laser pulse employed in each case was varied, considering the absorption of each sample to reach a similar level of carriers in both film (65 μJ/cm^2^) and cavity (112 μJ/cm^2^). The photobleaching signals observed for either bare NPL films at the exciton spectral position, or for the NPL cavity at lower and upper polariton energies, result from the depletion of their corresponding ground states caused by the intense excitation. In good agreement with their hybrid nature, cavity polaritons show a significantly shorter lifetime than the bare film exciton, as revealed by a detailed analysis of the photobleaching decay dynamics (Figure S19). Interestingly, the upper polariton exhibits a significantly longer lifetime (*≈* 17 ps) than the lower polariton (*≈*2 ps). This is a direct consequence of the specific design employed to build the cavity, with a detuning (≈310 meV) between the cavity mode at **k**_∥_ = 0 (≈2.48 eV) and the excitonic transition (2.79 meV), which provides a much stronger photon-like character to the lower polariton. The different contributions of the cavity and the 1s-1s excitonic transition to the formation of the lower and upper polariton are analyzed in detail in Figure S20, in which we represent the Hopfield coefficients attained by solving the two coupled oscillator Hamiltonian corresponding to the NPL cavity under analysis.

## Conclusions

In summary, this study presents an advanced colloidal synthesis protocol and ligand engineering of hybrid organic–inorganic MAPbBr_3_ NPLs, 11.3 ± 2.3 nm in lateral dimension and 1.7 ± 0.5 nm in thickness, to enhance their environmental and colloidal stability while preserving bright, narrow emission. Zwitterionic ligand-exchanged NPLs retained their morphology and optical properties for months. As a result of their high degree of monodispersity in all three dimensions, NPLs stack via their basal planes, forming extended SLs. High tendency of plane-to-plane assembly was also apparent in the colloidal state, translating into PL characteristics. In addition, nonmonotonic temperature evolution of PL energy revealed particularly enhanced exciton–phonon interaction driven by strong anisotropic confinement and motion of the organic cation. A better understanding of NPLs’ self-assembly guided us to control the extent of cluster formation and enabled the optical characterization of a single MAPbBr_3_ NPL. At cryogenic temperatures, individual MAPbBr_3_ NPLs exhibited emission narrowing down to an fwhm of 11 meV, and antibunched emission with g^(2)^(0) of 0.2, evidencing, for the first time, that the atomically precise hybrid organic–inorganic LHP NPLs may function as quantum emitters. Finally, we have shown that films of NPL assemblies are amenable for integration into Fabry-Pérot optical cavities, supporting exciton-polaritons whose dispersion relation displays a significant 200 meV Rabi splitting, outperforming or equaling previous results attained for other low-dimensional perovskites displaying quantum size effects, and providing a means to modify their absorption and emission properties controllably.

Further efforts in the realm of LHP NPLs should be directed toward the less understood compositions so far, e.g., FAPbX_3_ and AZPbX_3_ NPLs. A persistent challenge remains the stabilization of LHP NPLs to a degree comparable to that of their 3D NC counterparts, which can be achieved through the development of ligand chemistry or protective shells. We expect that the current findings will facilitate further integration of NPLs into optoelectronic and photonic applications.

## Methods

### Safety Statement

No unexpected or unusually high safety hazards were encountered.

### Precursor Stock Solution

Methylammonium bromide MABr (0.4 mmol, 45 mg), PbBr_2_ (0.4 mmol, 147 mg), and MLA (1.6 mmol, 215 mg) were dissolved in 10 mL of anhydrous dimethylformamide (DMF). The resulting transparent solution was filtered through a 0.2 μm filter and stored in an inert atmosphere.

### Synthesis of MAPbBr_3_ NPLs

Ligand-assisted reprecipitation (LARP) synthesis was adopted from ref.^66^ OLAm (0.12 mmol, 39.6 μL, distilled) was added under vigorous stirring to 2 mL of precursor stock solution, and the resulting precipitate was vortexed for approximately 10 min. The resulting white suspension (250 μL) was added dropwise to 8 mL of toluene, and the solution was stirred for 2–4 h at room temperature, gradually changing from colorless to pale green with bright blue PL. The crude solution was then centrifuged at 10000 rpm (13780 rcf) for 5 min, and the precipitate was discarded. The supernatant was washed with 0.5 volume equiv of dimethyl carbonate (DMC) followed by centrifugation at 12100 rpm (20130 rcf) for 3 min. The resulting NPLs were redispersed in 0.5–1 mL of anhydrous toluene.

### Ligand Exchange

To perform a ligand exchange, ca. 0.5 mL of NPL colloid in toluene is treated with the stock solution of the desired ligand. For example, for the ligand exchange with lecithin, 5–15 μL of 0.13 M lecithin stock solution in hexane was added; for the ligand exchange with C_8_C_12_–PEA, 2–10 μL of 0.12 M C_8_C_12_–PEA stock solution in mesitylene was added; for the ligand exchange with DDAB, 2–5 μL of 0.025 M DDAB stock solution in toluene was added; for the ligand exchange with 5k–PS-PEA, 15–30 μL of 0.05 M 5k–PS-PEA stock solution in toluene was added. The treated solution was stirred for 30 min, then washed with DMC or hexane in the case of 5k–PS-PEA, and the final precipitate was redispersed in anhydrous toluene.

### Microscopy Characterization

TEM and STEM images were collected with a JEOL JEM 2200FS electron microscope operating at an accelerating voltage of 200 kV. Image analysis was performed using ImageJ. HAADF-STEM images were recorded with a probe-corrected FEI Titan Themis operated at 300 kV with a convergence semi-angle of 18 mrad using a beam current of about 1 pA to minimize radiation damage.

### Atomic Force Microscopy

The topography images and height profiles of MAPbBr_3_ NPL films were recorded with Park NX10 AFM in noncontact mode (NCM). The images were processed using Gwyddion software.

### NMR Study

The NPLs precipitated after the ligand exchange were redispersed in *ca.* 0.5 mL of toluene-d_8_ and used for the NMR measurements of colloidal solutions. The digested samples were obtained by adding DMSO-*d*_6_ to the colloidal solutions.

Proton spectra were acquired at 16.4 T (700 MHz) using a 5 mm BBI probe connected to an Avance III HD spectrometer (Bruker). 64 scans per spectrum were acquired, and spectra were referenced to the 2H signal of the deuterated solvent. *T*_1_ relaxation times were determined using an inversion recovery experiment prior to the measurements, and the recycle delay was set to 5 times the slowest relaxing component, typically the solvent.

NOESY spectra were acquired using a phase-sensitive pulse sequence with gradient pulses during the mixing time.^102,103^ 256 slices in the indirect dimension were recorded with a recycle delay of 2 s, and mixing times were varied between 50 and 500 ms.

Phosphorus spectra were acquired at 11.7 T (500 MHz) using a 5 mm BBO ProdigyCryo probe with an Avance III HD spectrometer. Spectra were acquired using a 30° excitation pulse with power-gated proton decoupling and a recycle delay of 2 s. 8192 scans were acquired per intact NPL sample, while 256 scans per sample were acquired for decomposed NPLs.

### Powder X-ray Diffraction

Powder X-ray diffraction (XRD) patterns were collected on drop-cast films in a transmission mode with a STOE STADI P diffractometer. A Ge (111) monochromator (Cu–Kα radiation, λ = 1.54056 Å) and a Dectris MYTHEN 1K detector were employed for the measurements.

### WAXTS Analysis

WAXTS measurements were performed under ambient conditions at the XRD1 beamline of the Elettra Sincrotrone Trieste, using the setup of the GIWAXS measurements. NPLs colloidal suspensions were loaded inside borosilicate glass capillaries (Ø = 0.7 mm); 1D radial patterns were extracted from the 2D images upon azimuthal integration. Along with the samples’ scattering patterns, the pure solvent (toluene) signals were collected to be added as a blank trace to the 1D pattern model based on the Debye Scattering Equation (DSE).^104^ The scattering from the empty capillary and the sample environment were independently collected in the same experimental conditions to be subtracted from the 1D signal of the samples. Transmission measurements were performed to estimate the transmittance of the samples and correct the data for X-ray absorption using locally developed routines.

The reduced WAXTS data were analyzed using the Rietveld approach included in the TOPAS-R suite of programs (TOPAS V3.0, 2005, Bruker AXS, Karlsruhe, Germany). Starting from the split-cubic model later detailed, the unit cell parameters were relaxed resulting in an expansion along [001], the NPLs’ thickness direction, confirming the size-induced tetragonal distortion of the cubic structure (*P4/mmm*), and refined unit cell with c/a values ranging in a small interval (1.0322–1.0345) for the different samples. In contrast, previously analyzed MAPbBr_3_ NCs (unpublished data) displayed a cubic structure and metric with a = 5.92149(7) Å, as also found for the corresponding bulk material (a = 5.93076(2) Å).^73^

A “split-cubic” model was used for both MAPbBr_3_ NPLs and NCs structures, in which the disorder of Br^–^ anions about their “ideal” position (1/2,0,0 and equivalent ones) is modeled by splitting each one into four equiprobable sites with 1/4 occupancy.^105^ We have reported the same structural model for other hybrid organic-inorganic LHP NCs, such as FAPbBr_3_ and AzPbBr_3_ NCs.^106–108^ This model was also applied to bulk MAPbBr_3_.^104,109^ In the present study, the disorder of Br^–^ anions was first refined on MAPbBr_3_ NCs data at the 1/2,x,0 site, with x = 0.0681(3). The corresponding Pb–Br–Pb angle is 164.49(7)°. The coordinates of the disordered organic molecule reported for bulk MAPbBr_3_ were used.^73^ While refining this split model against the WAXTS data of NPLs, only the unit cell parameters were relaxed. The detected tetragonal distortion of the lattice results in a very small deviation (∼0.5°) of the axial and equatorial Pb–Br–Pb angles from the split-cubic value.

The morphological model was optimized by the DSE analysis, performed by Debussy,^104^ relying on a population of square prisms with two refinable NC sizes (L_a_ = L_b_ defining the NPLs’ basal plane, and L_c_ in the normal, thinner direction) and the relative size dispersions, described by a bivariate log-normal distribution function. The atomistic model of NPLs was calculated using the Rietveld-refined structure.

### GIWAXS Characterization

The samples for GIWAXS measurements were prepared on SiN_*x*_ membranes (Agar Scientific, Norcada) by means of a drying-mediated approach, whereby the NPL colloidal solution (ca. 25 μL) was slowly evaporated over a tilted support.

GIWAXS measurements were performed at the XRD1 beamline of the Elettra Sincrotrone Trieste. All measurements were performed under ambient conditions. The X-ray beam energy was set at 12.4 keV (λ = 1.00 Å), with the Pilatus 2 M detector (DECTRIS Ltd., Baden, Switzerland) positioned 300 mm away from the sample and a beam size of 200 × 200 mm^2^. 2D GIWAXS images were collected using a grazing incidence angle α_i_ = 0.2° and 10 s of exposure time. All GIWAXS images were analyzed using the GIDVIS software.^110^

### Ex Situ Absorption and PL Spectroscopy

UV–vis absorption spectra of NPLs dispersed in toluene were collected with a Jasco V770 spectrometer in a transmission mode. Photoluminescence (PL) spectra were measured with a Horiba Fluoromax-4P spectrometer equipped with a photomultiplier tube and a 150 W xenon lamp with an excitation wavelength of 400 nm. PLQY was measured employing a Hamamatsu Quantaurus-QY spectrometer with an excitation wavelength of 380 nm.

### In Situ Absorption Measurements

LARP synthesis of MAPbBr_3_ NPLs was conducted in a 25 mL custom-made flask equipped with an indentation with a path length of 1 mm to probe absorbance in case of high concentrations.^111^ The absorption spectra were measured with an Ocean Optics deuterium-tungsten light source (DH-2000-BAL-TTL-24 V) and an Ocean Optics HDX-XR broadband spectrometer. Custom-developed Python scripts were employed for processing and analysis.

### Ensemble Optical Measurements

Colloidal solution of lecithin-capped MAPbBr_3_ NPLs was drop-cast onto a crystalline Si wafer with a thermal oxide layer of 2 μm thickness. This sample was mounted inside an evacuated cryostat with a closed-loop liquid helium system, first cooled down to a target temperature of 4 K, and the temperature was adjusted for temperature-dependent measurements. NPL ensemble film was excited by the pulsed laser with a repetition rate of 5 MHz, at 405 nm (LDH-D-C-405, PicoQuant). Excitation power density was 0.01 μJ/cm.^2^ PL spectra were recorded with an EMCCD coupled to a monochromator (Princeton Instruments).

### Single-Particle Optical Measurements

The sample was prepared by diluting lecithin-capped MAPbBr_3_ NPL colloid solution (0.9 mg/mL) by a factor of 100 in anhydrous toluene, then diluting once more by a factor of 4 in a 3-mass% solution of polystyrene in toluene. 40 μL of final solution was spin-coated onto a glass coverslip (for RT measurement) and onto a crystalline Si wafer with a 2 μm-thick thermal oxide layer (for cryo-T measurement). A custom-built μ-PL setup was used for single-particle spectroscopy. Samples were mounted on XYZ nanopositioning stages (Smaract for RT; Attocube for cryo-T). For cryo-T measurement, the sample and the stage were placed inside a cryostat, which was evacuated and cooled down to a target temperature of 4 K with a closed-loop helium system. Single NPLs were excited using a diode laser at 405 nm (LDH-D-C-405, PicoQuant) with a repetition rate of 10 MHz, coupled to an optical fiber. Laser beam was focused on the sample by a microscope objective (oil-immersive, NA = 1.3, 100x for RT; air, NA = 0.8, 100x for cryo-T) to excite single NPLs. Power densities were 40 nJ/cm^2^ for RT and 20 nJ/cm^2^ for cryo-T measurements. NPL emission was collected by the identical objective lens, passed through a long-pass filter with a cutoff at 430 nm, and then directed into a monochromator-coupled EMCCD (Princeton Instruments) to record the spectra. Photon statistics were collected using a Hanbury-Brown-Twiss setup consisting of two APDs, 50/50 beam splitter, and a TCSPC module (PicoHarp 300, PicoQuant). Time-resolved PL traces of single NPLs were recorded by one of the APDs.

### Assembled NPL Film and Cavity Preparation

The NPL solution (8 mg/mL in a hexane/octane mixture, 2:1) was drop-cast inside a nitrogen-filled glovebox onto two types of substrates: low-fluorescence glass (used for reference NPL films) and substrates consisting of 200 nm of silver coated with 15 nm of sputtered silicon nitride (Fraunhofer) for cavity assembly. Prior to deposition, all substrates were cleaned in an ultrasonic bath with 2% Hellmanex solution, followed by sequential rinsing with acetone and 2-propanol. Following NPL deposition, a 30 nm thick silver mirror was thermally evaporated under high vacuum (10^–6^ mbar) using a Univex 250 system (Leybold Vacuum). The evaporation was carried out at a rate of 1 Å/s, with the thickness continuously monitored by a quartz crystal microbalance to ensure precise control.

### Optical Absorptance Characterization and Optical Constants Determination

Polarization and angular-dependent measurements were conducted with a double goniometer configuration (Universal Measurement Accessory, UMA) attached to a UV–vis-NIR spectrophotometer (Cary 5000, Agilent). Absorptance (A) was attained as A = 1 – R – T, where R and T stand for reflectance and transmittance, respectively. The optical constants for the assembled NPL film were estimated by fitting the experimental T and R measured at different angles of incidence and polarizations using the method developed by Forouhi and Bloomer.^112^

### Ultrafast Spectroscopy Analysis

Ultrafast transient absorption spectroscopy measurements were performed using a pump-and-probe setup. The signal generated by a Yb:KGW pulsed laser is split into two beams. One is directed to an optical parametric amplifier (Orpheus, Light Conversion), where either 400 nm (for the NPL assembly) or 440 nm (for the NPL cavity) pump pulses with a narrow spectral width (4 nm) were generated. The other beam passes through a delay line and, subsequently, a sapphire crystal to generate broadband probe pulses. The signal was collected using a CMOS detector attached to a spectrometer (HELIOS, Ultrafast Systems) in a reflection configuration, with an angle of incidence and collection of the probe beam of 26 °. The HELIOS Fire software produces a 3D wavelength-delay time-ΔA matrix, where ΔA is the result of subtracting the absorbance of the nonexcited sample from that of the photoexcited one, thus representing a differential absorbance $\Delta A=-\log\left(\frac{R_{\mathrm{exc}}}{R_{0}}\right)$ .

### Optical Simulations

Optical reflectance, transmittance, and absorptance, as well as electric field intensity and absorption profiles, are calculated with a MATLAB code using a transfer matrix method based on the Abeles formalism.^113^ The thickness of each layer is determined experimentally using a profilometer, and the refractive indices are either taken from the bibliography for Ag, SiN_*x*_, or modeled through the fitting of the experimental R and T spectra using a Forouhi-Bloomer model of the dielectric constant for NPL film.

## Supplementary Material







## Notes

Video S1: In situ absorption spectroscopy of MAPbBr_3_ NPL growth during the initial stages of reaction progress (AVI)
Video S2: In situ absorption spectroscopy of MAPbBr_3_ NPL growth over the whole reaction time scale (AVI)
Employed materials, syntheses of precursors and NCs, in situ absorption spectroscopy, MAPbBr_3_ NPL characterization (TEM and optical studies), NPL stability studies, structural WAXS analysis, MAPb­(Br/Cl)_3_ and CsPbBr_3_ NPLs characterization, PL properties of MAPbBr_3_ NPLs and respective nanocubes at the ensemble and single-particle levels, spin-coated MAPbBr_3_ NPL films, and exciton-polariton formation measurements (PDF)
