# Supplementary material for: Ligand-Engineered Methylammonium Lead Bromide Nanoplatelets: Single-Photon Emission and Strong Light-Matter Coupling
Source: ACS Nano. 2026 Mar 31;20(14):11358–73. doi: 10.1021/acsnano.6c01048 (PMC13085852; doi:10.1021/acsnano.6c01048)
Supplement: Supplementary file 3 [file nn6c01048_si_003.pdf]

Supporting Information

Ligand-Engineered Methylammonium Lead Bromide Nanoplatelets: Single-Photon Emission and Strong Light-Matter Coupling

Taras V. Sekh,[1,2] Taehee Kim,[1,2] Sebastian Sabisch, [1,2] Federica Bertolotti,[3] Laura Caliò,[4] Juan F. Galisteo-López,[4] Francisco J. Coto-Ruiz,[4] Lucía Santiago-Andrades,[4] Antonietta Guagliardi,[5] Norberto Masciocchi,[3] Rolf Erni,[6] Hernán Miguez,[4] Gabriele Rainò,[1,2] Maryna I. Bodnarchuk,[1,2*] Maksym V. Kovalenko[1,2*]

[1] Institute of Inorganic Chemistry, Department of Chemistry and Applied Biosciences, ETH Zürich, 8093 Zürich, Switzerland

[2] Laboratory for Thin Films and Photovoltaics, Empa−Swiss Federal Laboratories for Materials Science and Technology, 8600 Dübendorf, Switzerland

[3] Department of Science and High Technology and To.Sca.Lab, University of Insubria, via Valleggio 11, 22100 Como, Italy

[4] Instituto de Ciencia de Materiales de Sevilla, Consejo Superior de Investigaciones Científicas-Universidad de Sevilla, Sevilla 41092, Spain

[5] Istituto di Cristallografia and To.Sca.Lab, Consiglio Nazionale delle Ricerche, via Valleggio 11, 22100 Como, Italy

[6] Electron Microscopy Center, Empa−Swiss Federal Laboratories for Materials Science and Technology, CH-8600 Dübendorf, Switzerland

[*]corresponding authors: mvkovalenko@ethz.ch, maryna.bodnarchuk@empa.ch

Table of contents

MATERIALS......3
Figure S1. In-situ recorded optical absorption spectroscopy of $MAPbBr_3$ NPLs during the growth......5
Figure S2. Capping ligands for $MAPbBr_3$ NPLs......6
Figure S3. Stability towards sequential purification of lecithin-capped $MAPbBr_3$ NPLs......7
Figure S4. Environmental stability of $MAPbBr_3$ NPLs......8
Figure S5. WAXTS studies on $MAPbBr_3$ NPLs......9
Figure S6. $MAPb(Br/Cl)_3$ NPLs......10
Figure S7. $CsPbBr_3$ NPLs......11
Figure S8. Low-angle WAXTS studies on $MAPbBr_3$ NPLs......12
Figure S9. C_8C_{12}-PEA treatment of NPLs......13
Figure S10. PL properties of $MAPbBr_3$ NPL films with different NPL concentrations......14
Figure S11. Temperature-dependent PL of $CsPbBr_3$ NPLs......15
Figure S12. PL properties of cubic $MAPbBr_3$ NCs......16
Figure S13. Temperature-dependent PL of lecithin-capped $MAPbBr_3$ NPLs......17
Figure S14. PL properties of single cubic $MAPbBr_3$ NC......18
Figure S15. Spin-coated $MAPbBr_3$ NPL films......19
Figure S16. Optical properties of cavity materials......20
Figure S17. Exciton-polariton energy dispersion relation for a NPL optical cavity......21
Figure S18. Spatial and spectral distribution of the optical field intensity inside the NPL cavity......22
Figure S19. Decay dynamics of the photobleaching signal......23
Figure S20. Relative exciton and photon contribution to the polaritons: Hopfield coefficients......24
References......25

MATERIALS

Chemicals

CsBr (99.9%, Aldrich), $PbBr_2$ (99.999%, Aldrich), methylamine (40wt% in MeOH, TCI), hydrobromic acid (HBr, 48wt% in water, Acros), 2-octyl-1-dodecanol (97%, Aldrich), ω-hydroxyterminated polystyrene (M_n=5000, Polymer Source, Inc.), triethylamine (TEA, 99.5%, Aldrich), phosphorous (V) oxychloride ($POCl_3$, 99%, Aldrich), ethanolamine (99.5%, Aldrich), acetic acid (99.8%, Aldrich), trioctylphosphine oxide (TOPO, 90%, Strem), methylamine acetate (Greatcell Solar Materials), bis(2,4,4-trimethylpentyl)phosphinic acid (BTPPA, 90%, Fluorochem), L(-) malic acid (MLA, 97%, Strem), oleylamine (OLAm, min. 95%, Strem, distilled), oleic acid (90%, Aldrich), N,N-dimethylformamide (DMF, 99.8%, extra dry, ThermoFisher), dimethyl carbonate (DMC, 99%, Aldrich), lecithin (97%, Roth), didodecyldimethylammonium bromide (DDAB, 98%, Aldrich), hexane (anhydrous, 95%, Aldrich), n-octane (99%, Roth, for synthesis), toluene (anhydrous, 99.8%, Aldrich), tetrahydrofuran (THF, 99.5%, extra dry, ThermoScientific), acetone (99.5%, Aldrich).

Methylammonium bromide (MABr) synthesis (adopted from Ref.[1])

40wt% methylamine in methanol (20 mL), 48wt% HBr in water (15 mL) were mixed in 100 mL of ethanol and stirred for 2 h in an ice bath under Nitrogen atmosphere. Crystallization of MABr was achieved employing a rotary evaporator at 40-50 °C, followed by washing the product three times with diethyl ether and drying it at 50 °C overnight under vacuum.

C_8C_{12}-phosphoethanolamine (PEA) synthesis (adopted from Ref.[2])

Solution of 2-octyl-1-dodecanol (0.025 mol, 7.5 g) in 25 mL of THF along with TEA (0.0275 mol, 3.83 mL) was added dropwise upon vigorous stirring into a solution of $POCl_3$ (0.03 mol, 2.78 mL) in 2.5 mL THF in an ice water bath. The reaction mixture was subsequently kept at 20 °C for 15 min to complete the reaction. Next, ethanolamine (0.03 mol, 1.81 mL) and TEA (0.06 mol, 0.8 mL) in 37.5 mL of THF were added dropwise under vigorous stirring to the reaction mixture kept in a room-temperature water bath. Subsequently, the mixture was heated to 40 °C for 15 min to complete the ring closure. Finally, the reaction mixture was filtered to remove precipitated triethylamine hydrochloride, and the filtrate solution is dried. An oily residue was dissolved in a mixture of 5.7 mL of acetic acid and 2.6 mL of distilled water at 70 °C. After 30 min the product was separated by beating with 125-150 mL of acetone. After cooling to 10 °C, the product was collected and dried overnight under vacuum at 40–50 °C.

5k-polystyrene (PS)-PEA synthesis (adopted from Ref.[2])

Solution of ω-hydroxyterminated polystyrene with M_n=5000 (0.2 mmol, 1 g) in 0.2 mL of THF along with TEA (0.22 mmol, 30 μL) was added dropwise upon vigorous stirring into a solution of $POCl_3$ (0.24 mmol, 22 μL) in 20 μL THF in an ice water bath. The reaction mixture was subsequently kept at 20 °C for 15 min to complete the reaction. Next, ethanolamine (0.24 mmol, 15 μL) and TEA (0.48 mmol, 7 μL) in 0.3 mL of THF were added dropwise under vigorous stirring to the reaction mixture kept in a room-temperature water bath. Subsequently, the mixture was

heated to 40 °C for 15 min to complete the ring closure. Finally, the reaction mixture was filtered to remove precipitated triethylamine hydrochloride, and the filtrate solution is dried. An oily residue was dissolved in a mixture of 45 µL of acetic acid and 20 µL of distilled water at 70 °C. After 30 min the product was separated by beating with 1-1.2 mL of acetone. After cooling to 10 °C, the product was collected and dried overnight under vacuum at 40–50 °C.

$PbBr_2$-TOPO precursor, 0.04M

Lead (II) bromide (1 mmol, 367 mg) and TOPO (5 mmol, 1.93 g), were dissolved in 5 ml n-octane at 110 °C on a hotplate in air, cooled to room temperature, diluted in 20 ml of n-hexane and filtered through a 0.22 µm PTFE syringe filter.

MA-BTPPA-OA precursor, 0.067M

Methylamine acetate (0.6 mmol, 55 mg), BTPPA (3 mL), and oleic acid (2 mL) were added into 5 mL of n-octane and heated to 60°C in air until the salt dissolution.

$MAPbBr_3$ nanocubes (adopted from Ref.[3])

$PbBr_2$-TOPO precursor (500 µL, 0.04M) was diluted with 3 mL of n-hexane in an open flask on a stirring plate. To this solution, MA-BTPPA-OA precursor (77µL, 0.067M) was swiftly injected. Immediately, C_8C_{12}-PEA ligand solution (5 mg in 50 µL of mesitylene) was added. NCs were purified by the addition of 3 equiv. of antisolvent (ethylacetate:acetonitrile, 2:1 v:v), centrifugation at 12100 rpm (20130 rcf) for 1 min, and redispersion of the precipitate in n-octane. The purification was repeated two times in total and the colloid was dissolved in 0.5 mL of n-octane, followed by a centrifugation at 12100 rpm (20130 rcf) for 1 min.

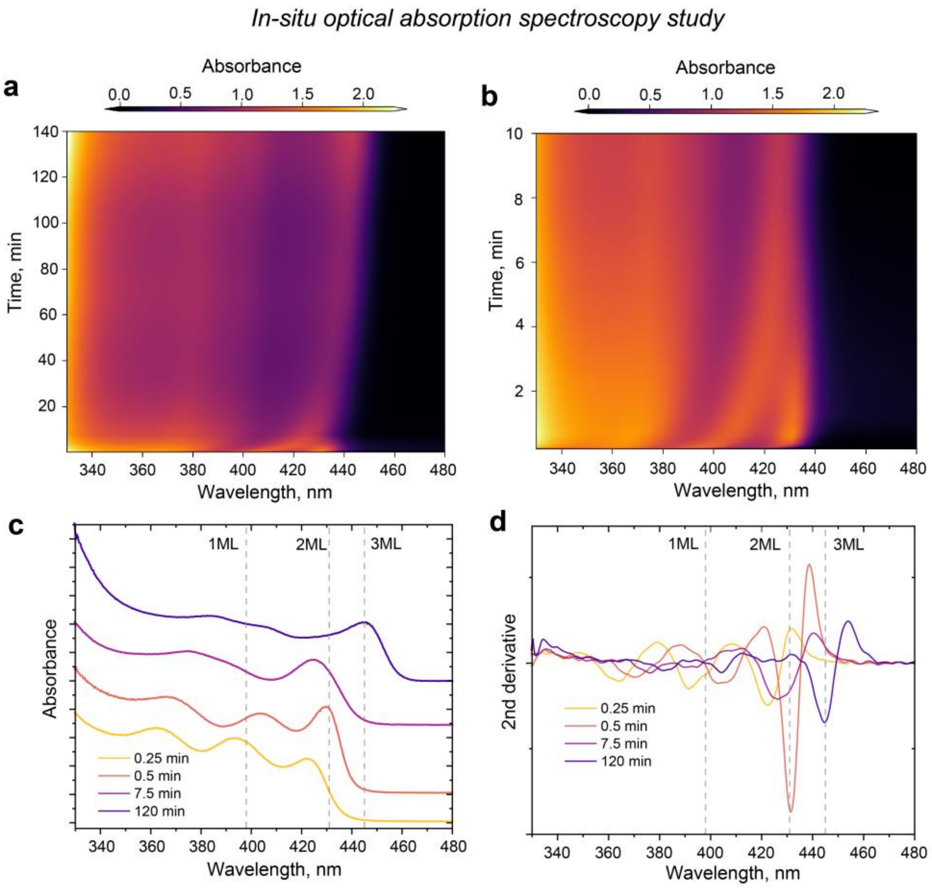

Figure S1. In-situ recorded optical absorption spectroscopy of $MAPbBr_3$ NPLs during the growth. (a, b) 2D absorption plots corresponding to longer (140 min) and shorter (10 min) growth time windows, respectively. (c, d) Absorption profiles and their corresponding second derivatives at selected times. The reference literature values for 1 ML, 2 ML, and 3 ML $MAPbBr_3$ NPLs are indicated with grey dashed lines.[4-6]

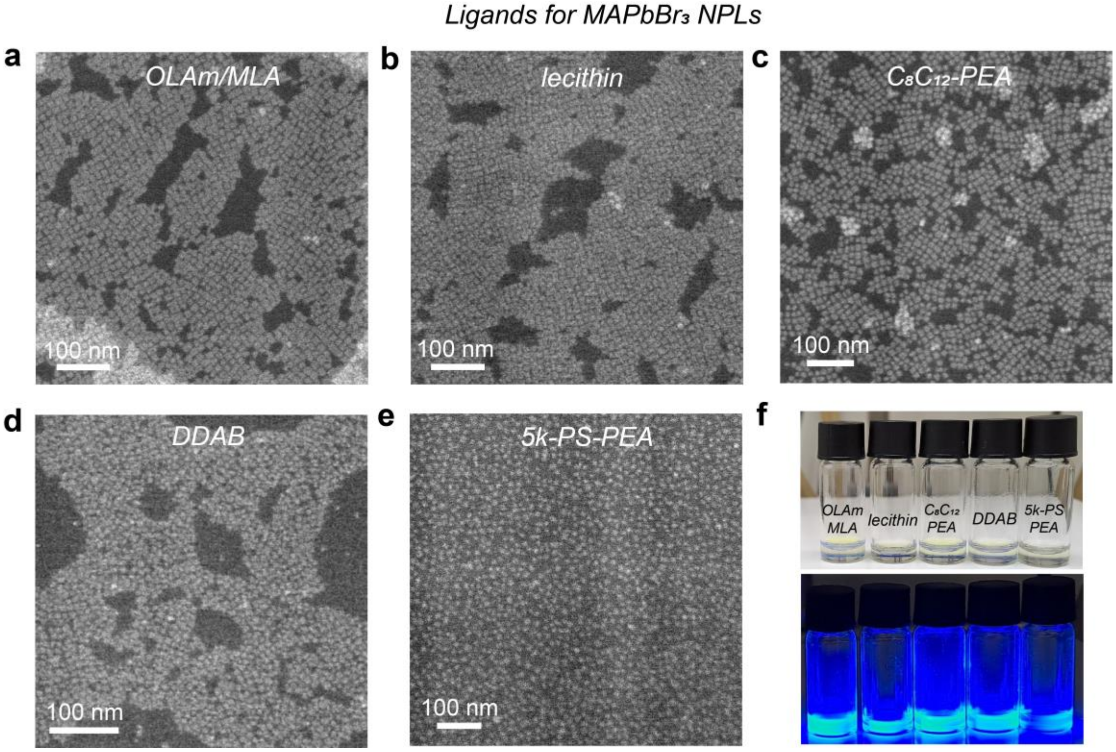

Figure S2. Capping ligands for $MAPbBr_3$ NPLs. (a-e) DF-STEM images of $MAPbBr_3$ NPLs capped with (a) pristine OLAm and MLA ligands, (b) lecithin, (c) C_8C_{12}-PEA, (d) DDAB, and (e) 5k-PS-PEA. (f) The appearance of $MAPbBr_3$ NPL colloidal solutions under ambient light (above) and UV irradiation (below). From left to right, the vials are filled with colloidal $MAPbBr_3$ NPLs capped with OLAm and MLA, lecithin, C_8C_{12}-PEA, DDAB, and 5k-PS-PEA, respectively.

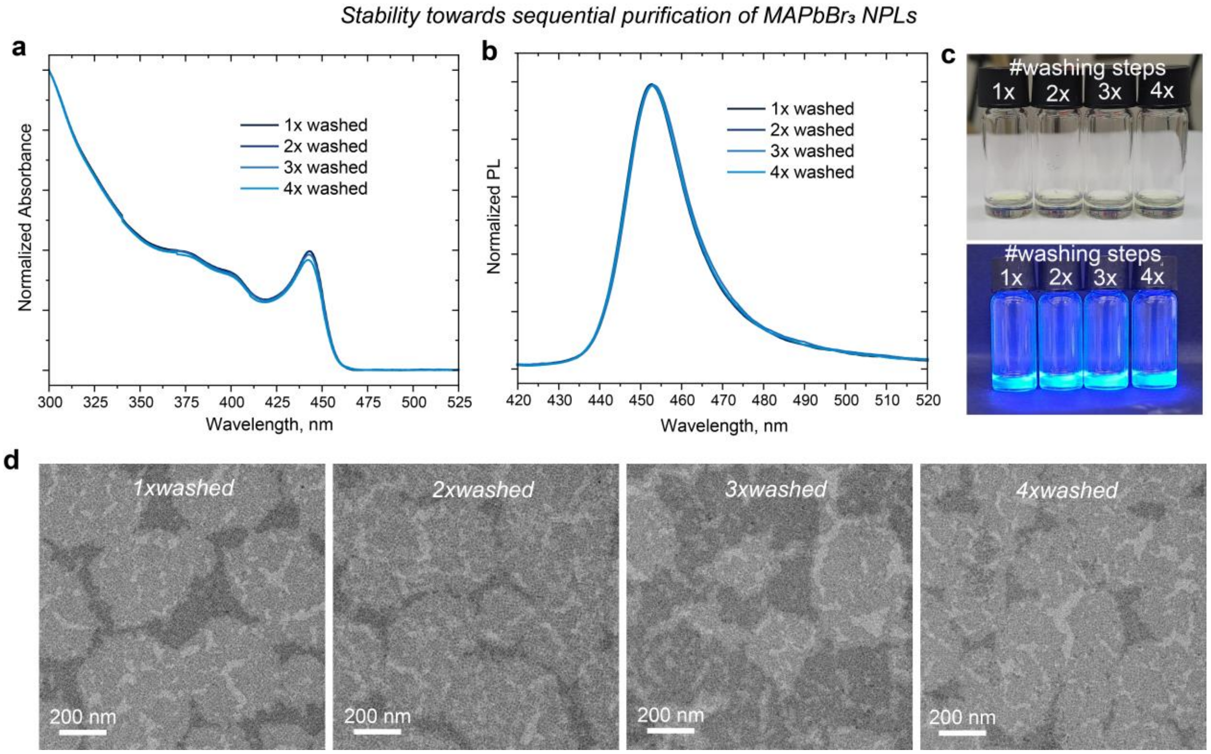

Figure S3. Stability towards sequential purification of lecithin-capped $MAPbBr_3$ NPLs. (a) PL and absorption spectra of $MAPbBr_3$ NPLs washed with DMC for different numbers of times. No shifts in both absorption and PL spectra are observed. (b) The appearance of $MAPbBr_3$ NPL colloidal solutions washed sequentially under ambient light (above) and UV irradiation (below). From left to right, the vials with NPLs washed 1x, 2x, 3x, and 4x times, respectively. (d) BF-STEM images of $MAPbBr_3$ NPL washed different numbers of times. Overall, the NPLs demonstrate exceptional stability during sequential washings, likely due to the enhanced stability of NPL clusters in the colloidal solution and the non-aggressive antisolvent (DMC) employed.

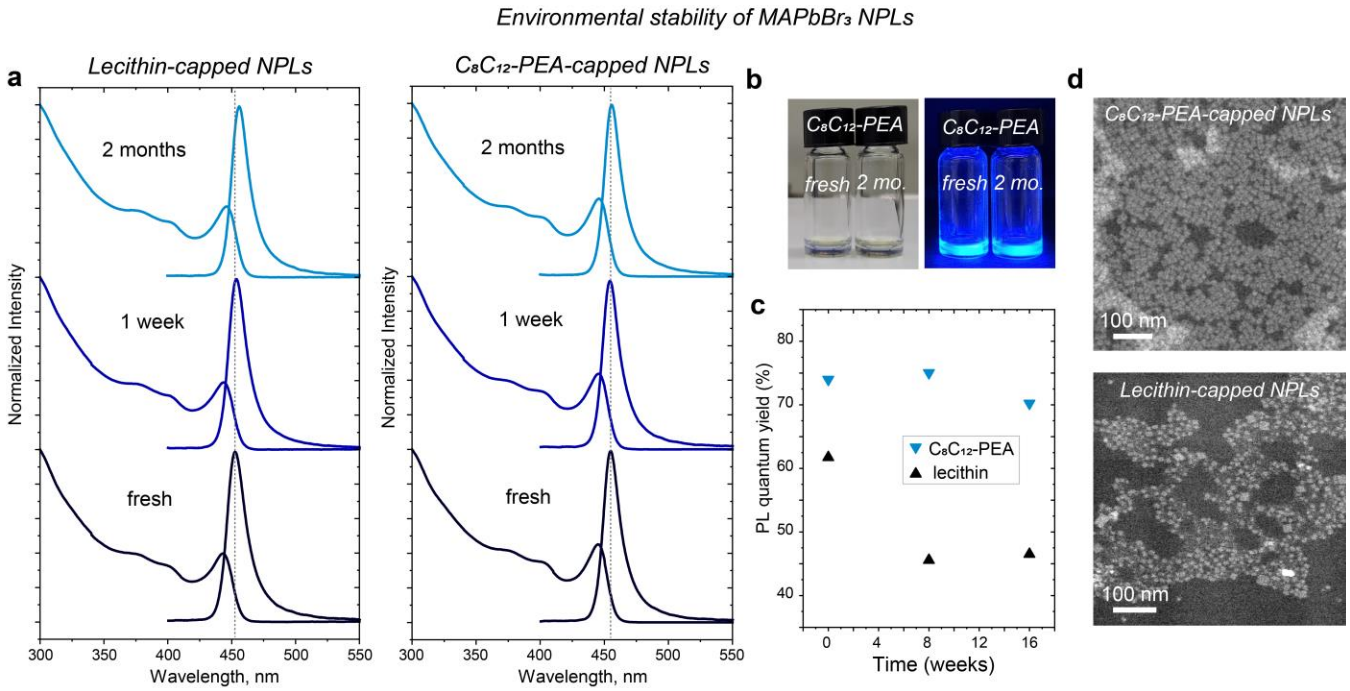

Figure S4. Environmental stability of $MAPbBr_3$ NPLs. (a) Absorption and PL spectra of lecithin-capped (left) and C_8C_{12}-PEA-capped (right) $MAPbBr_3$ NPL colloidal solutions with different times of air exposure – freshly synthesized NPLs, after 1 week, and after 2 months. C_8C_{12}-PEA-capped NPLs demonstrate no shift in absorption and PL spectra over a few months' storage, whereas lecithin-capped NPLs exhibit a shift of ca. 3 nm upon prolonged air exposure. (b) The appearance of C_8C_{12}-PEA-capped $MAPbBr_3$ NPL colloidal solutions as freshly prepared (left vial) and after 2 months' storage (right vial) under ambient light and UV irradiation. (c) PLQY values of colloidal solutions for C_8C_{12}-PEA-capped and lecithin-capped NPLs at different storage times. (d) DF-STEM images of $MAPbBr_3$ NPLs capped with C_8C_{12}-PEA (above) and lecithin (below) after 4 months' storage, revealing C_8C_{12}-PEA-capped NPLs staying intact and lecithin-capped NPLs partially merged into larger NCs.

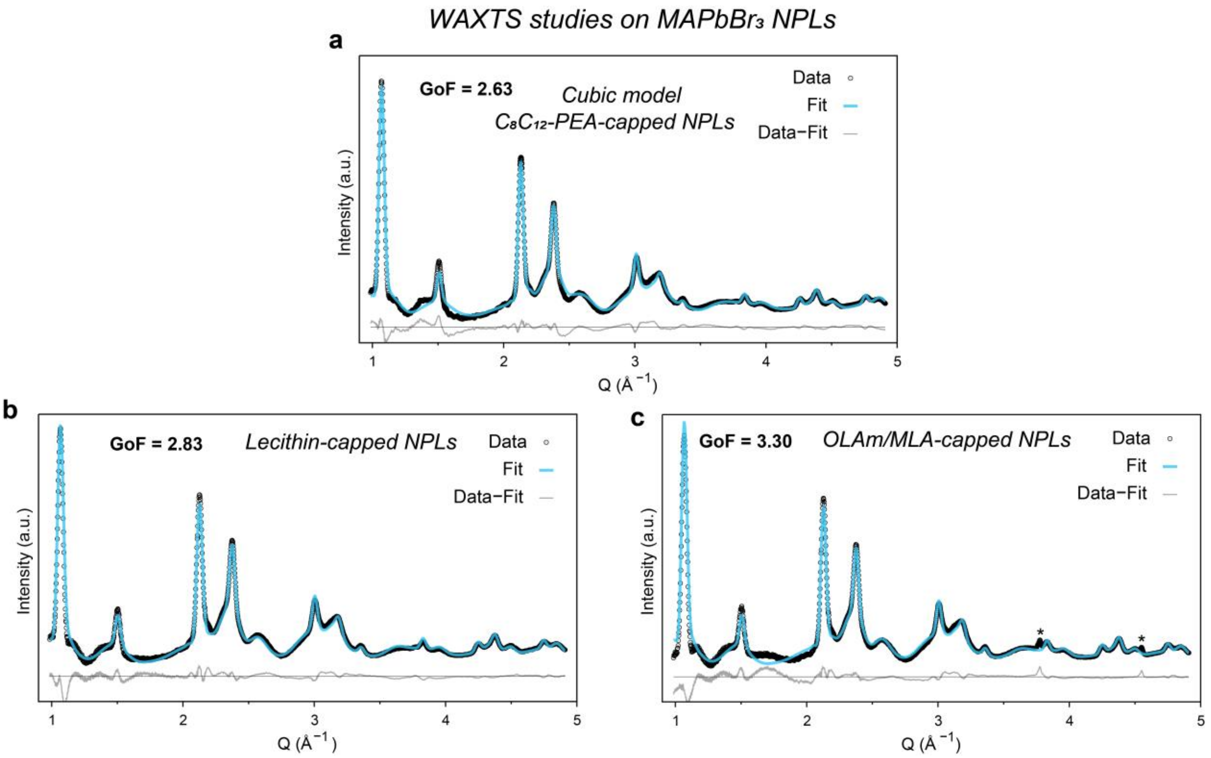

Figure S5. WAXTS studies on $MAPbBr_3$ NPLs. (a) The DSE best fit of the solvent-subtracted colloidal C_8C_{12}-PEA-capped NPLs data obtained with the split cubic atomistic model combined with a cubic unit cell (a = 5.903(1) Å), highlighting a slightly worse match with respect to the tetragonal unit cell model in Figure 1e (GoF = 2.62 vs 2.13). (b) DSE best fit of the solvent-subtracted lecithin-capped colloidal NPLs data obtained according to the split-model and a tetragonal unit cell with parameters: a = b = 5.920(2) Å; c= 6.122(5) Å; c/a = 1.0341(9). Refined number-based sizes: L_a = 9.97 nm, σ/L_a = 22%; L_c= 1.62 nm, σ/L_c = 28% (GoF = 2.83). (c) DSE best fit of the solvent-subtracted OLAm/MLA-capped colloidal NPLs data (same tetragonally-distorted structural model as in b). Refined unit cell parameters: a = b = 5.916(2) Å; c = 6.123(6)Å; c/a = 1.035(1). Refined number-based sizes: L_a = 11.30 nm, σ/L_a = 20 %; L_c = 1.69 nm, σ/L_c = 27% (GoF = 3.30). Additional tiny peaks appearing due to the misplaced beamstop during the measurement are marked with an asterisk.

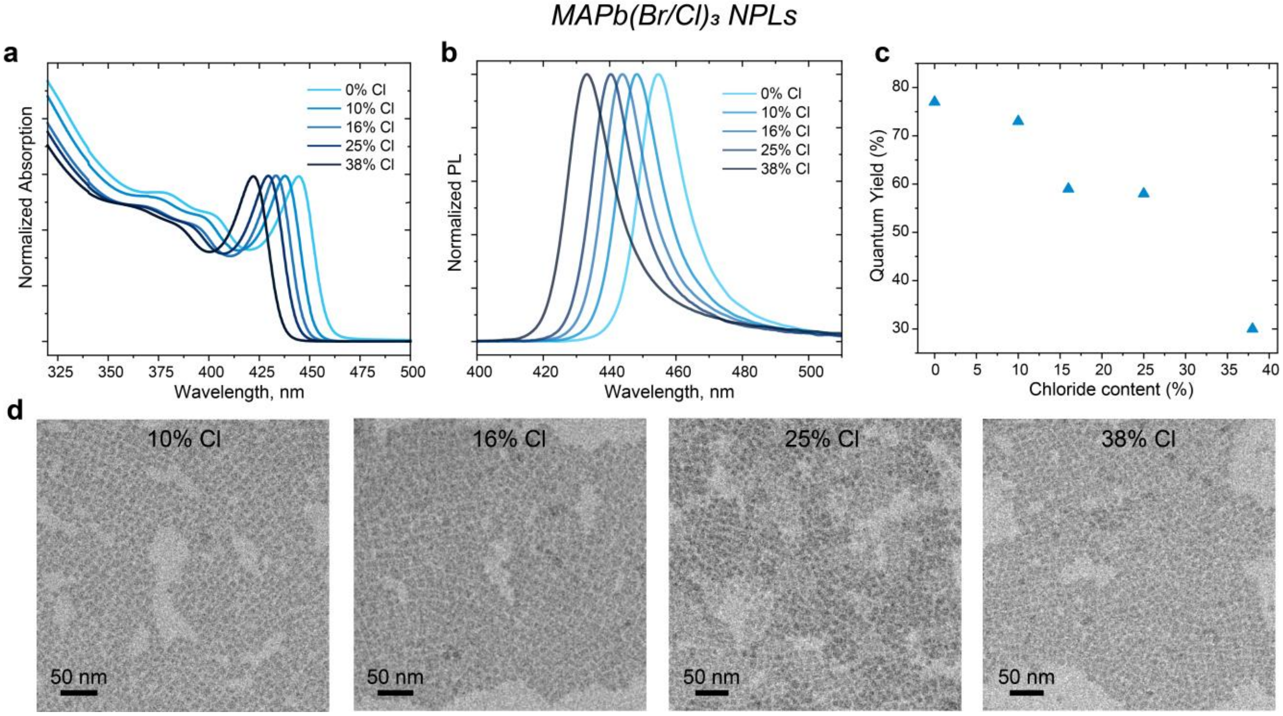

Figure S6. MAPb(Br/Cl)$_3$ NPLs. (a) Absorption and (b) PL spectra of MAPb(Br/Cl)$_3$ NPL colloidal solutions with different halide composition, revealing tunable optical properties of NPLs. (c) Dependence of colloidal solutions PLQY on Cl content. PLQY values remain high (~60%) at the Cl percentage of 25%, while upon further increase in Cl content, PLQY drops. (d) BF-STEM images of MAPb(Br/Cl)$_3$ NPLs with different halide compositions demonstrating that the NPLs stay intact even at high Cl content.

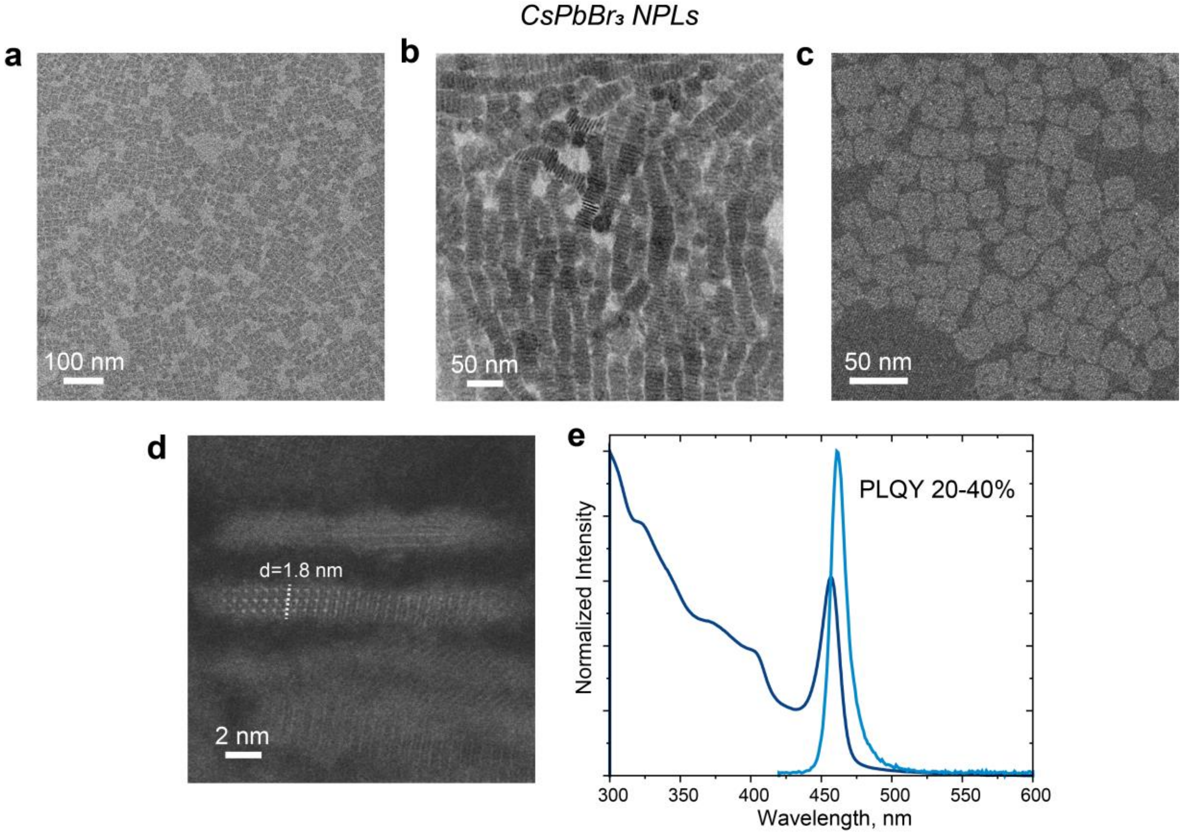

Figure S7. $CsPbBr_3$ NPLs. (a, b) BF-STEM images of $CsPbBr_3$ NPLs in a monolayer and an assembly, respectively. Similar to $MAPbBr_3$ counterparts, $CsPbBr_3$ NPLs are prone to forming assemblies of stacked face-to-face NPLs. (c) HAADF-STEM of a $CsPbBr_3$ monolayer. (d) HAADF-STEM image of a vertically standing $CsPbBr_3$ NPL demonstrating three unit cells in a thickness direction (d=1.8 nm). (e) Absorption and PL spectra of a $CsPbBr_3$ NPL colloidal solution in toluene.

Low-angle WAXTS data on $MAPbBr_3$ NPLs

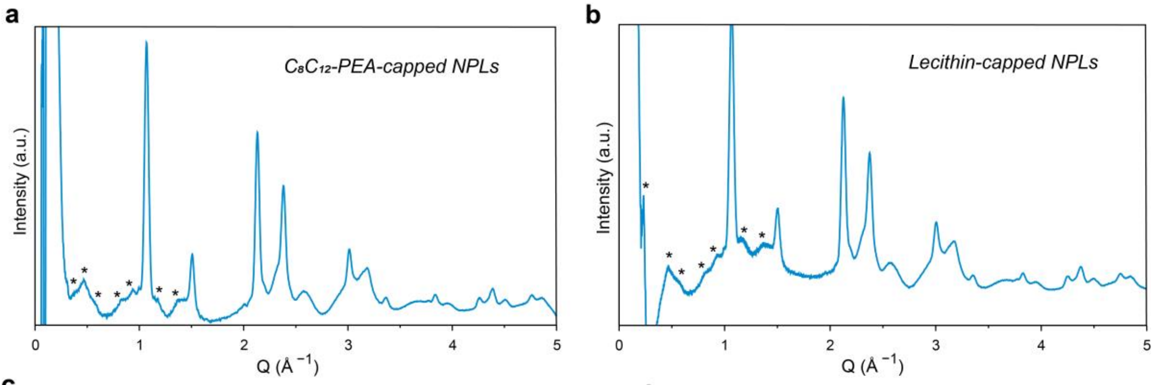

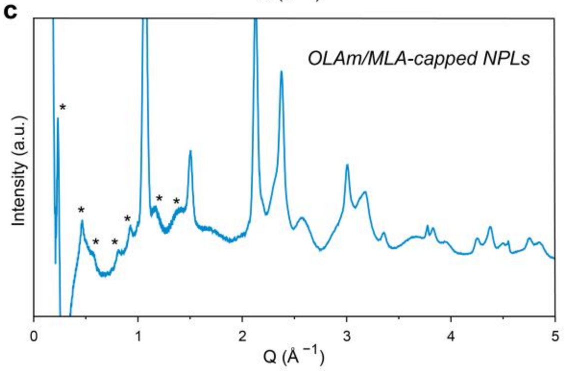

d

SLs reflections

Q (Å⁻¹)	d (Å)	n
0.232	27.08	2
0.464	13.54	4
0.580	10.83	5
0.812	7.74	7
0.928	6.77	8
1.160	5.42	10
1.392	4.51	12

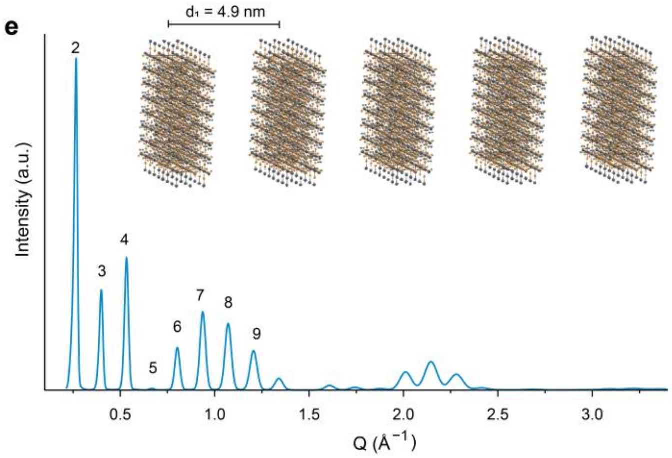

Figure S8. Low-angle WAXTS studies on $MAPbBr_3$ NPLs. (a-c) WAXS patterns including low-angle region for C_8C_{12}-PEA-capped, lecithin-capped, and OLAm/MLA-capped $MAPbBr_3$ NPLs, respectively. Measured data are from toluene suspensions, shown data are solvent-subtracted. The SL reflections in the low-angle region are marked with an asterisk. (d) The list of SLs reflections with the indicated d-spacings and diffraction order n. e) The simulated 1D sequence of XRD peaks calculated for a 4.9 nm stacking (d_1) of 1.8 nm thick NPL, where $q_n = 2\pi n/d_1$, with a few n values inserted in the plot.

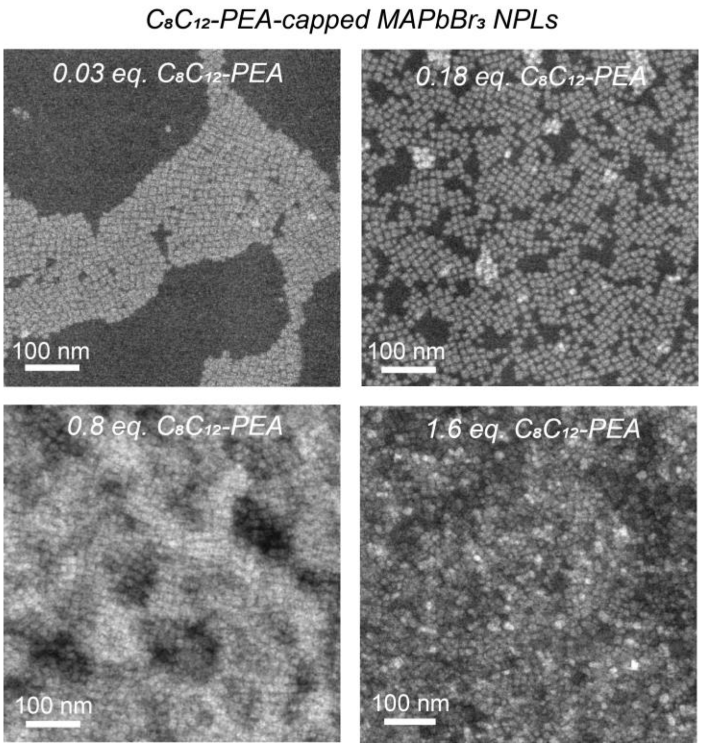

Figure S9. C_8C_{12}-PEA treatment of NPLs. DF-STEM images of $CsPbBr_3$ NPLs after treatment with different amounts of C_8C_{12}-PEA – 0.03, 0.18, 0.8, 1.6 equivalents of C_8C_{12}-PEA with respect to OLAm. Merged and degraded NPLs start to appear already at 0.8 eq. of C_8C_{12}-PEA, signifying that possible is only partial replacements of pristine ligands.

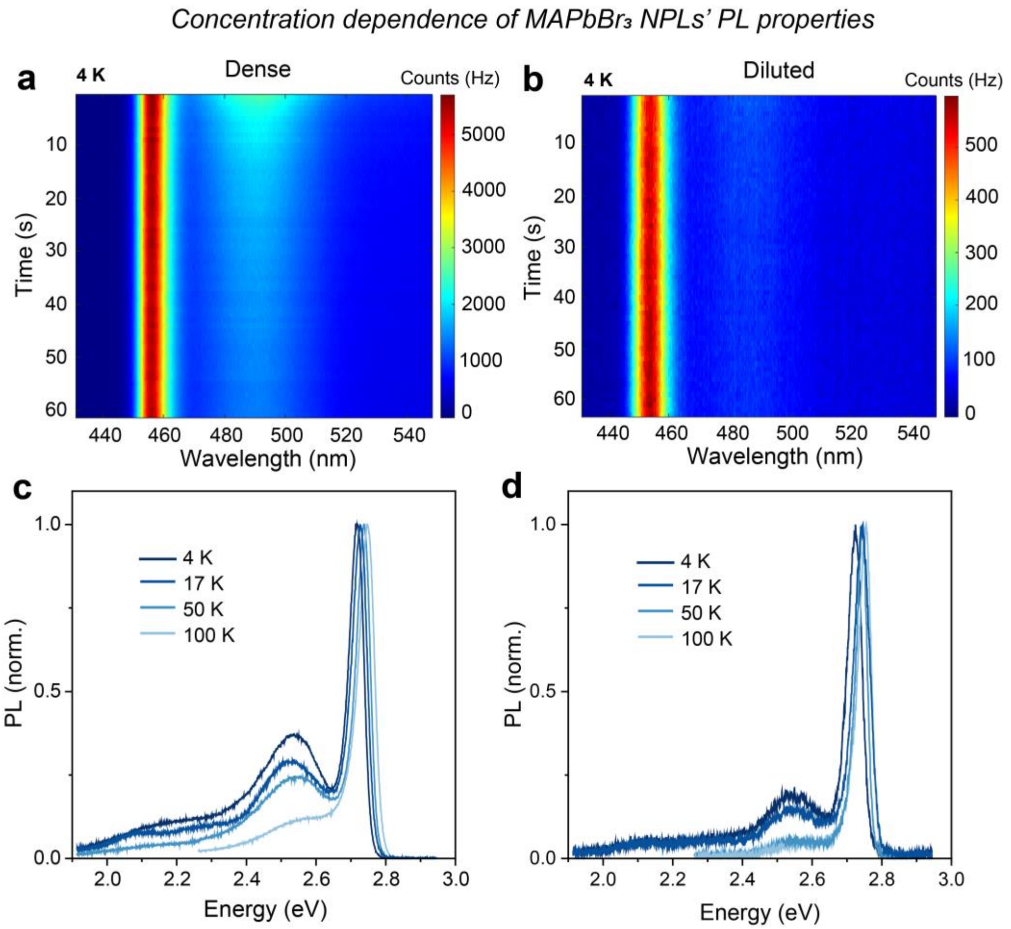

Figure S10. PL properties of $MAPbBr_3$ NPL films with different NPL concentrations. (a,b) PL time-series at 4 K for dense (2 mg/ml) and diluted (0.02 mg/ml) films, respectively. (c,d) Normalized PL spectra at different temperatures (4-100 K) for dense and diluted films, respectively.

Temperature-dependent PL of CsPbBr₃ NPLs

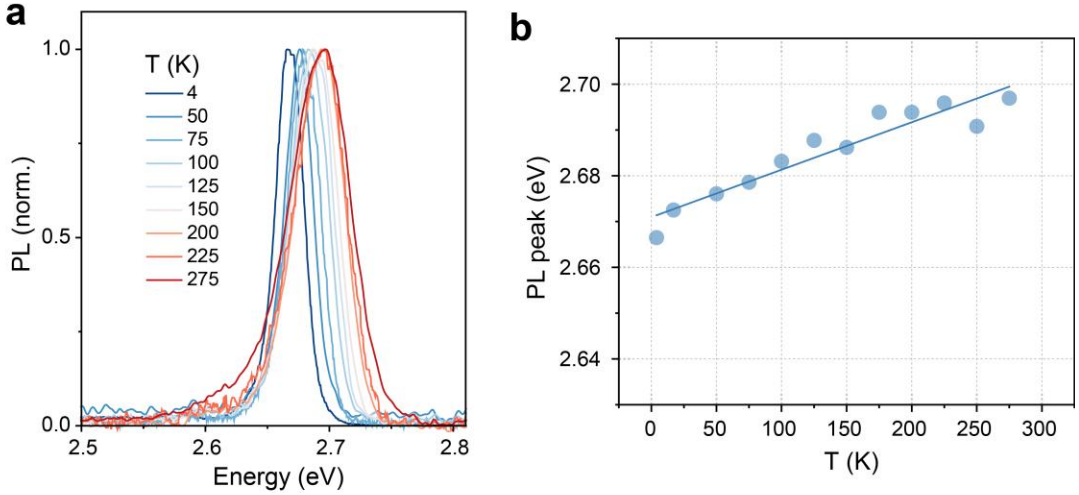

Figure S11. Temperature-dependent PL of $CsPbBr_3$ NPLs. (a) Normalized PL spectra of $CsPbBr_3$ NPLs (4 ML) at varying temperatures. (b) Temperature-dependent PL peak energy fitted with Bose-Einstein single-oscillator model, yielding A_{TE} = 0.1 meV/K and A_{EP} ~ 0.

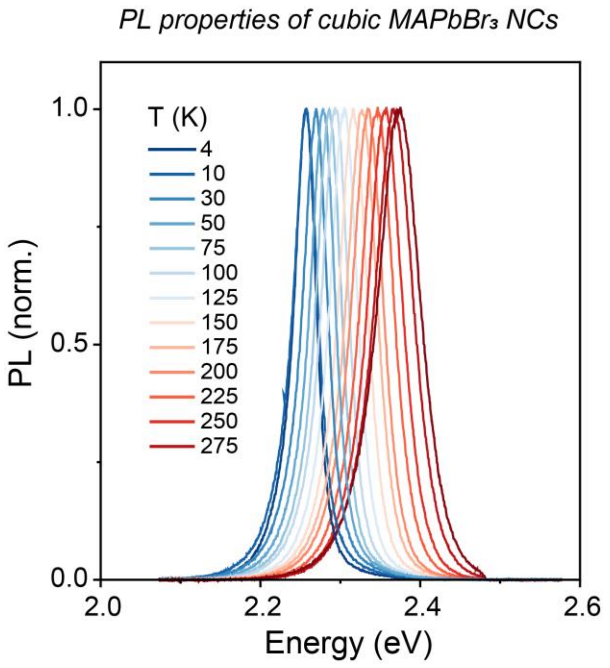

Figure S12. PL properties of cubic $MAPbBr_3$ NCs. Normalized PL spectra of $MAPbBr_3$ cubic NC with an edge length of 6 nm at varying temperatures (4-275 K).

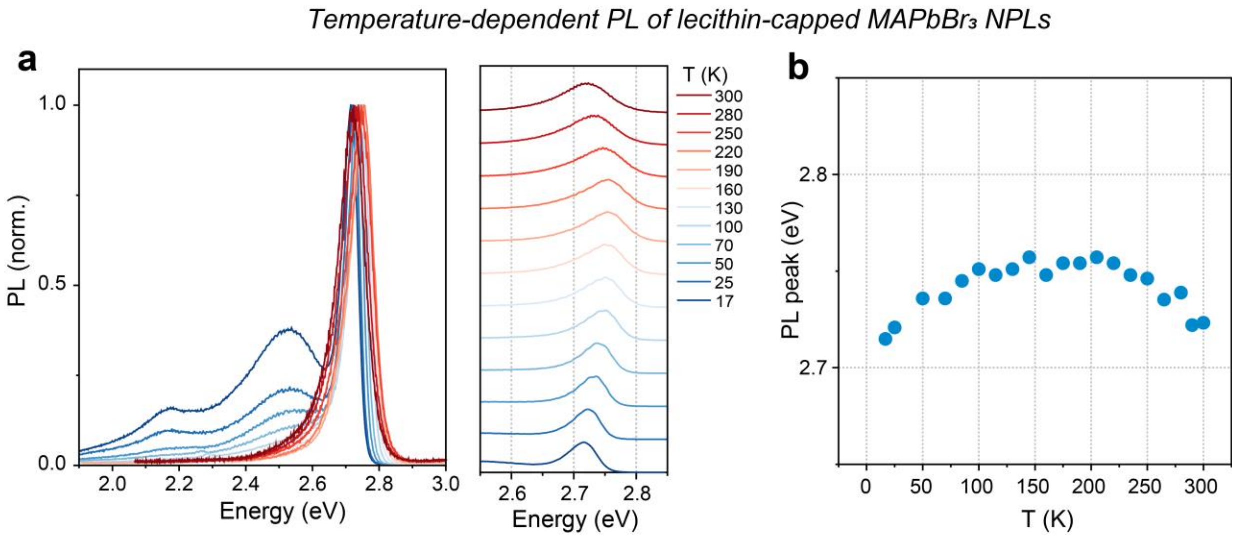

Figure S13. Temperature-dependent PL of lecithin-capped $MAPbBr_3$ NPLs. (a) Left: normalized PL spectra of lecithin-capped $MAPbBr_3$ NPL film at varying temperatures, 17-300 K. Right: magnified excitonic emission peak showing non-monotonous peak energy evolution across temperatures. (b) Temperature evolution of PL peak energy of lecithin-capped $MAPbBr_3$ NPLs (3 ML thick) depicted with blue dots.

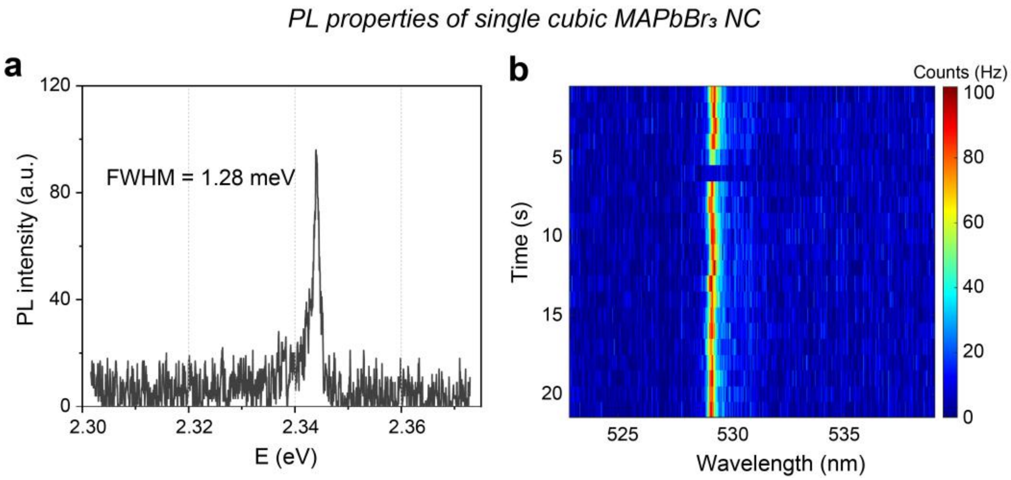

Figure S14. PL properties of single cubic $MAPbBr_3$ NC. (a) PL spectrum of a single $MAPbBr_3$ cubic NC with an edge length of 6 nm at 4 K. (b) PL time-series of the corresponding single NC.

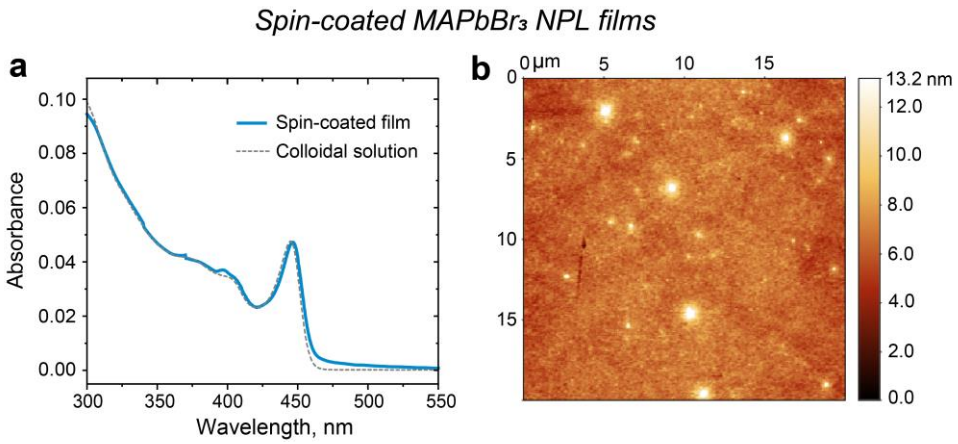

Figure S15. Spin-coated $MAPbBr_3$ NPL films. (a) Absorption spectra of the spin-coated film and corresponding colloidal solution depicted with blue and grey dashed lines, respectively. (b) AFM image of spin-coated film.

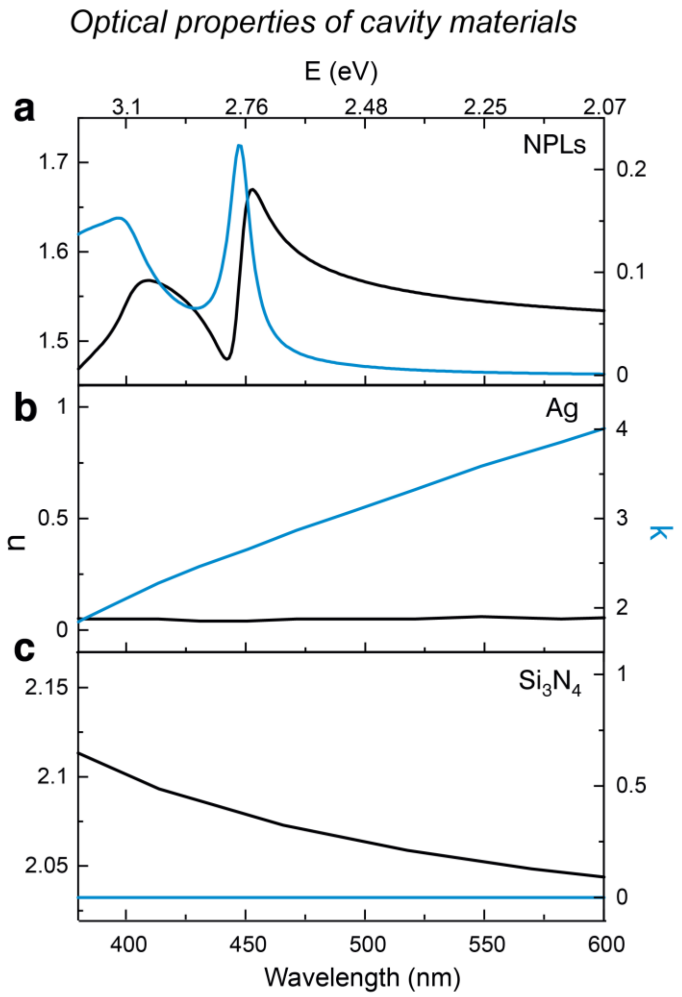

Figure S16. Optical properties of cavity materials. Optical constants for (a) NPL assembly film (upper panel), (b) Silver (middle panel), and (c) Silicon Nitride (lower panel).

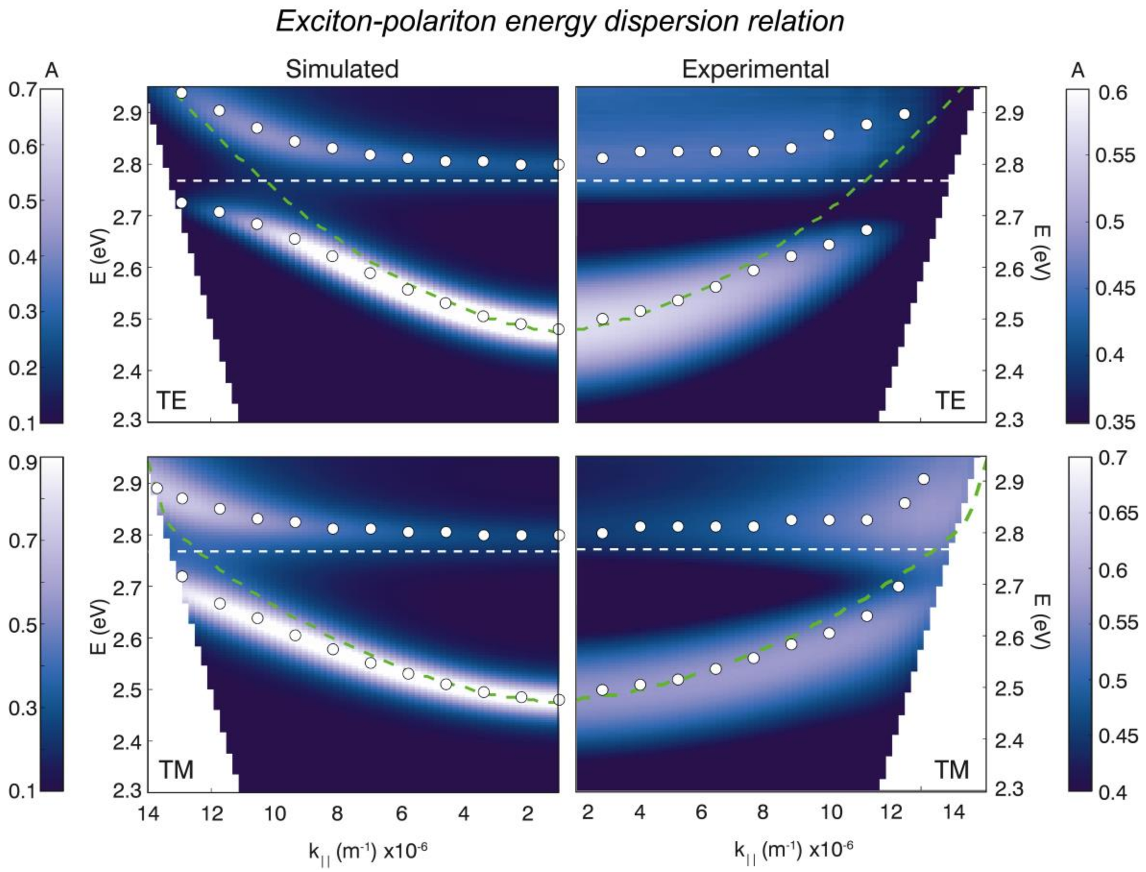

Figure S17. Exciton-polariton energy dispersion relation for a NPL optical cavity. Theoretical (left panels) and experimental (right panels) absorptance energy dispersion maps for TE (upper panels) and TM (lower panels) polarizations of the incident beam. White dots indicate the spectral position of the experimental absorption maxima, horizontal dotted white lines indicate the positions of the 1s-1s excitonic transitions, and green dotted lines represent the underlying cavity mode dispersion.

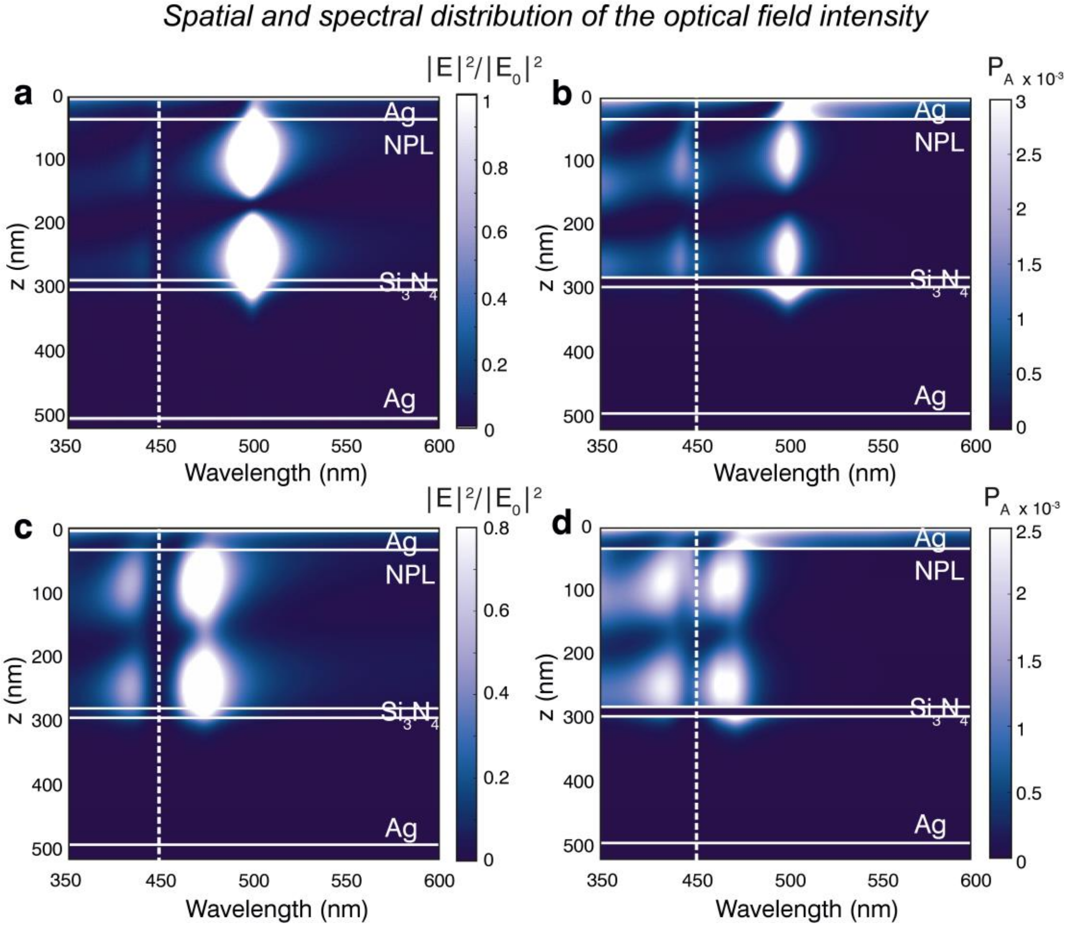

Figure S18. Spatial and spectral distribution of the optical field intensity inside the NPL cavity. TMM calculations were used to attain the spatial and spectral profiles of (a,c) the electric field intensity, $|\mathbf{E}(\mathbf{r})|^2$, and (b,d) the absorbed luminous power, P_A, for the NPL cavity, for an angle of incidence 0° (a,b) and 50° (c,d). Calculations are performed considering a plane wave impinging on the top silver mirror (position 0 in the z-axis) and propagating along the z-direction. Interfaces between layers are indicated by horizontal white lines, while vertical white dashed line represent the position of the 1s-1s excitonic transition. This representation allows to visualize the order of the photon resonance participating in the coupling (the 2nd order, in this case) and its splitting through the number of nodes observed in the spatial profiles of both $|\mathbf{E}(\mathbf{r})|^2$ and P_A.

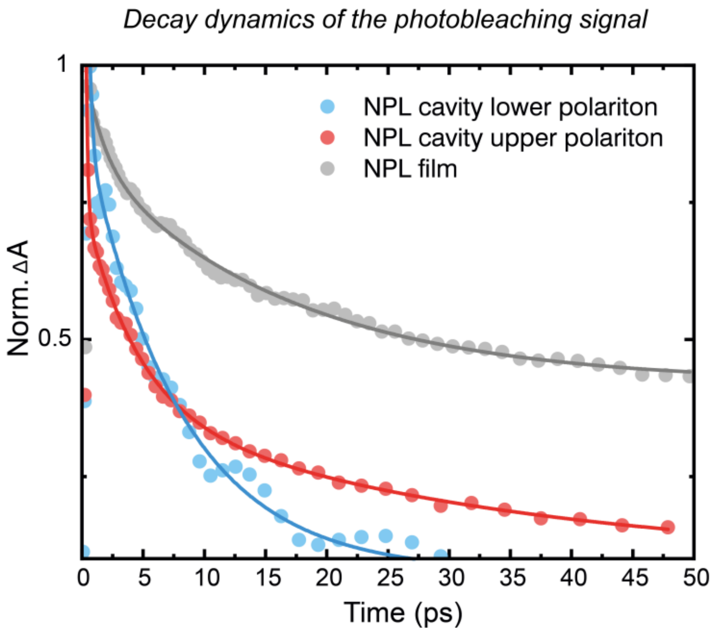

Figure S19. Decay dynamics of the photobleaching signal. Decay dynamics of the photobleaching signal measured with transient absorption spectroscopy corresponding to the ground state bleach of the NPL film exciton (grey scatters), and the NPL cavity upper and lower polaritons (red and blue scatters, respectively). Fittings to multiple exponential functions are plotted with solid lines following the same colour code. From them, exciton and polariton lifetimes are obtained.

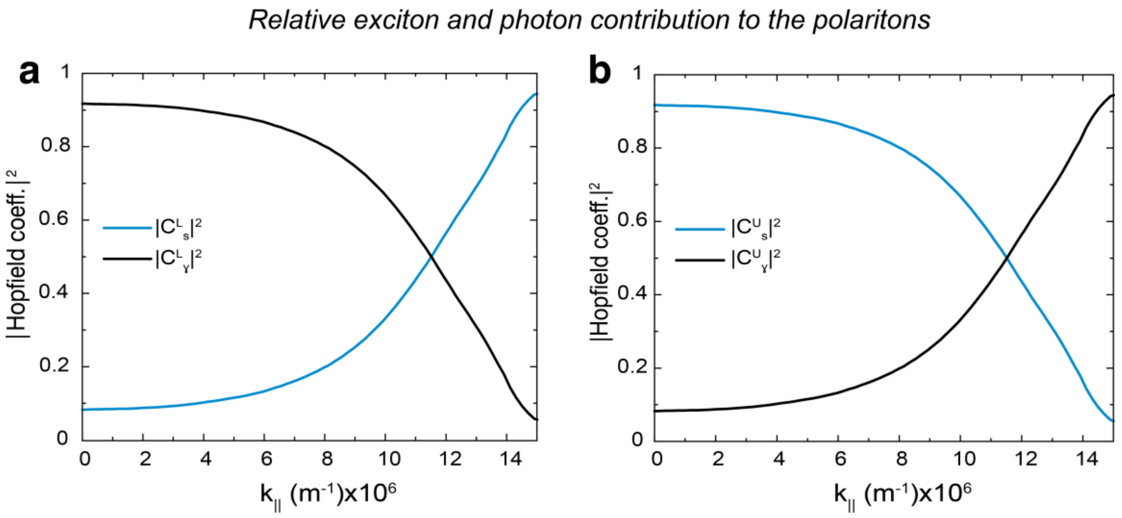

Figure S20. Relative exciton and photon contribution to the polaritons: Hopfield coefficients. The superposition of photon and exciton states leading to the formation of NPL exciton-polaritons can be analyzed by solving the simplified eigenvalue equation for the Tavis-Cummings Hamiltonian, $\hat{H}_{TC}$:

$$\hat{H}_{TC}\psi_{L,U} = \hbar\omega_{L,U}\,\psi_{L,U} \qquad \text{(S1)}$$

The new hybrid states, ψ_L, and ψ_U, are the wavefunctions of the lower and upper polariton states, which result from the hybridization of the cavity photon and the 1s-1s NPL exciton transition involved in the coupling:

$$\psi_{L,U} = C_\gamma^{L,U}\psi_\gamma + C_s^{L,U}\psi_s \text{ (S2)}$$

In equation (S2), C_i^j are the **Hopfield coefficients**, which fulfill the normalization condition:

$$\left|C_\gamma^j\right|^2 + \left|C_s^j\right|^2 = 1 \qquad \text{(S3)}$$

On this grounds, Eq.(S1) becomes:

$$\begin{pmatrix} \hbar\omega_\gamma & \hbar\frac{\Omega_{\gamma,s}}{2} \\ \hbar\frac{\Omega_{\gamma,s}}{2} & \hbar\omega_s \end{pmatrix}\begin{pmatrix} C_\gamma \\ C_s \end{pmatrix} = \hbar\omega_{L,U}\begin{pmatrix} C_\gamma \\ C_s \end{pmatrix} \text{ (S4)}$$

By solving this two coupled oscillator Hamiltonian, we attain the Hopfield coefficients $\left|C_i^j\right|^2$, which give us the degree of contribution of each state to the different exciton-polariton states observed, as shown in Figure S19 for the (a) lower and (b) upper polariton of the NPL cavity under analysis.

References

(1) Zhuo, S.; Zhang, J.; Shi, Y.; Huang, Y.; Zhang, B. Self-template-directed synthesis of porous perovskite nanowires at room temperature for high-performance visible-light photodetectors. *Angew. Chem., Int. Ed. Engl.* **2015**, *54*, 5693-5696.
(2) Morad, V.; Stelmakh, A.; Svyrydenko, M.; Feld, L. G.; Boehme, S. C.; Aebli, M.; Affolter, J.; Kaul, C. J.; Schrenker, N. J.; Bals, S.; et al. Designer phospholipid capping ligands for soft metal halide nanocrystals. *Nature* **2024**, *626*, 542-548.
(3) Akkerman, Q. A.; Nguyen, T. P. T.; Boehme, S. C.; Montanarella, F.; Dirin, D. N.; Wechsler, P.; Beiglbock, F.; Raino, G.; Erni, R.; Katan, C.; et al. Controlling the Nucleation and Growth Kinetics of Lead Halide Perovskite Quantum Dots. *Science* **2022**, *377*, 1406-1412.
(4) Weidman, M. C.; Goodman, A. J.; Tisdale, W. A. Colloidal Halide Perovskite Nanoplatelets: An Exciting New Class of Semiconductor Nanomaterials. *Chem. Mater.* **2017**, *29*, 5019-5030.
(5) Weidman, M. C.; Seitz, M.; Stranks, S. D.; Tisdale, W. A. Highly Tunable Colloidal Perovskite Nanoplatelets through Variable Cation, Metal, and Halide Composition. *ACS Nano* **2016**, *10*, 7830-7839.
(6) Wang, Q.; Liu, X.-D.; Qiu, Y.-H.; Chen, K.; Zhou, L.; Wang, Q.-Q. Quantum confinement effect and exciton binding energy of layered perovskite nanoplatelets. *AIP Advances* **2018**, *8*. 025108
